# The influence of AccD5 on AccD6 carboxyltransferase essentiality in pathogenic and non-pathogenic *Mycobacterium*

**DOI:** 10.1038/srep42692

**Published:** 2017-02-16

**Authors:** Jakub Pawelczyk, Albertus Viljoen, Laurent Kremer, Jaroslaw Dziadek

**Affiliations:** 1 Institute for Medical Biology, Polish Academy of Sciences, Lodz, Poland; 2 Centre National de la Recherche Scientifique FRE 3689, Centre d’études d’agents Pathogènes et Biotechnologies pour la Santé, Université de Montpellier, Montpellier, France; 3 INSERM, CPBS, 34293 Montpellier, France

## Abstract

Malonyl-coenzyme A (CoA) is a crucial extender unit for the synthesis of mycolic and other fatty acids in mycobacteria, generated in a reaction catalyzed by acetyl-CoA carboxylase. We previously reported on the essentiality of *accD6*_*Mtb*_ encoding the functional acetyl-CoA carboxylase subunit in *Mycobacterium tuberculosis*. Strikingly, the homologous gene in the fast-growing, non-pathogenic *Mycobacterium smegmatis* - (*accD6*_*Msm*_) appeared to be dispensable, and its deletion did not influence the cell lipid content. Herein, we demonstrate that, despite the difference in essentiality, *accD6*_*Msm*_ and *accD6*_*Mtb*_ encode proteins of convergent catalytic activity *in vivo*. To identify an alternative, AccD6-independent, malonyl-CoA synthesis pathway in *M. smegmatis*, a complex genetic approach combined with lipid analysis was applied to screen all five remaining carboxyltransferase genes (*accD1*-*accD5*) with respect to their involvement in mycolic acid biosynthesis and ability to utilize acetyl-CoA as the substrate for carboxylation. This approach revealed that AccD1_*Msm*_, AccD2_*Msm*_ and AccD3_*Msm*_ are not essential for mycolic acid biosynthesis. Furthermore, we confirmed *in vivo* the function of AccD4_*Msm*_ as an essential, long-chain acyl-CoA carboxyltransferase, unable to carboxylate short-chain substrate. Finally, our comparative studies unambiguously demonstrated between-species difference in *in vivo* ability of AccD5 carboxyltransferase to utilize acetyl-CoA that influences AccD6 essentiality in pathogenic and non-pathogenic mycobacteria.

Mycolic acids represent essential components of the *Mycobacterium tuberculosis* cell envelope. These long-chain (C_60–90_) β-hydroxy fatty acids with a shorter (C_24–26_) α-alkyl branch constitute up to 60% of the mycobacterial cell wall^1^,^2^,^3^,^4^. Playing a crucial role in envelope architecture and impermeability, these acids determine the natural resistance of mycobacteria to most antibiotics and represent key factors in mycobacterial virulence. Biosynthesis of mycolic acids requires two types of fatty acid synthase, FAS I and FAS II, which operate in unison in *de novo* synthesis and in the elongation of fatty acyl chains^5^. Despite the impressive progress made in understanding the structure and metabolism of mycolic acids over the past 15 years, there are still unresolved issues regarding the catalytic steps of the FAS machinery. Among these issues, the role and substrate specificity of the acyl carboxylases is still of great interest because of their importance in the process that leads to the synthesis of full-length, mature mycolates as well as other straight- and branched-chain fatty acids. Biotin-mediated carboxylation of short-chain fatty acyl coenzyme A (CoA) esters is a critical step in lipid metabolism^6^. This process consists of two catalytic steps: (i) the carboxylation of biotin to form carboxybiotin and (ii) the transfer of a carboxyl group from the biotin to a substrate. Each half-reaction is catalyzed by a specific carboxylase subunit: the first step by biotin carboxylase (α-subunit) and the second step by carboxyltransferase (β-subunit). Each subunit represents a single polypeptide encoded by a separate gene^7^. In *M. tuberculosis*, three genes encode potential biotin carboxylases, *accA1* to *accA3*, whereas six genes are believed to encode carboxyltransferases, *accD1* to *accD6*, and one gene encodes a regulatory ε-subunit (*accE5*)^8^. Because the β-subunits confer the substrate specificity of the holoenzyme, the unusual variety of carboxyltransferase (*accD*)-encoding genes reflects the ability of the mycobacterial carboxylases to transfer the carboxyl group on several distinct substrates. All *accD* family members have their clear homologues in *M. smegmatis* however one more gene (MSMEG_2169) demonstrating significant homology to *M. tuberculosis accD3* is present in this bacterium. There are at least three defined steps in the synthesis of fatty/mycolic acid requiring acyl carboxylation on specific substrates (Fig. 1). (i) The acetyl-CoA carboxylase catalyzes the carboxylation of acetyl-CoA in the initial and limiting reaction, providing malonyl-CoA that serves as a two-carbon extender unit, incorporated into the growing acyl chain during the repetitive FAS I/FAS II cycle^5^. (ii) The propionyl-CoA carboxylase activity allows for the synthesis of methylmalonyl-CoA, which is the building block for methyl-branched fatty acids biosynthesis^9^. (iii) The final step in the synthesis of full-length, mature mycolic acid relies on long-chain acyl-CoA carboxylase which activates the α-alkyl branch prior to condensation with meromycolyl-AMP^10^,^11^,^12^. Transposon site hybridization (TraSH) analysis and gene replacement experiments have suggested that among the six carboxyltransferases in *M. tuberculosis* AccD6 (Rv2247), AccD5 (Rv3280) and AccD4 (Rv3799c) are essential for cell viability^13^,^14^ and are expressed at high levels during mycolic acid biosynthesis^14^,^15^. Thus, in recent years, efforts have been made to identify the adequate carboxylase subunits able to transfer a carboxyl group on acetyl-, propionyl- or acyl-CoA.

Because of its central role in fatty acid metabolism, the identification of the mycobacterial carboxyltransferase that catalyzes malonyl-CoA synthesis has been the most important issue since the inception beginning of this research field. We previously provided the first evidence for *accD6* (Rv2247) essentiality and showed that *accD6* encodes a functional acetyl-CoA carboxyltransferase that supplies *M. tuberculosis* with malonyl-CoA^14^, thus confirming previous *in vitro* studies^15^. Unexpectedly, we also demonstrated that the homologous gene in the fast-growing, non-pathogenic *M. smegmatis* MSMEG_4329 (*accD6*_*Msm*_) is dispensable for mycobacterial growth and that its deletion did not influence the fatty/mycolic acid content, challenging its role in malonyl-CoA synthesis in this species^14^. *In vitro* biochemical analysis of AccD5 (Rv3280) revealed both an acetyl- and a propionyl-CoA substrate specificity with clear preference for the latter, emphasizing its putative engagement in methylmalonyl-CoA synthesis^16^. However, convincing *in vivo* studies are necessary to conclusively establish the functional role of AccD5 in mycobacteria. AccD4 (Rv3799c) remains the least characterized mycobacterial carboxyltransferase. A *Corynebacterium glutamicum* mutant devoid of an *accD4* homolog exhibited a “mycolate-less” phenotype and a lack of tetradecylmalonic acid, the predicted product of the long-chain acyl-CoA carboxylase, but retained the synthesis of fatty acids^11^. The *in vitro* ability of AccD4_*Mtb*_ to carboxylate long-chain (C_24_) acyl-CoA was subsequently reported but is still awaiting confirmation in mycobacterial cells^17^.

Herein, we conducted a thorough and complex genetic study to shed light on the actual *in vivo* substrate specificity of all six AccD enzymes.

## Results

### AccD6 from *M. smegmatis* and that from *M. tuberculosis* exhibit convergent *in vivo* activity despite their difference in essentiality

We previously demonstrated the essentiality of *accD6* (Rv2247) in *M. tuberculosis (Mtb*)^14^. It was also shown that AccD6_*Mtb*_ is a functional carboxyltransferase subunit of the malonyl-CoA-producing enzyme *in vivo*. Surprisingly, parallel studies on a non-pathogenic mycobacterial strain - *M. smegmatis (Msm*) - have unequivocally proved that the *accD6* homolog - MSMEG_4329 (*accD6*_*Msm*_) - is dispensable in this bacterium and that gene disruption has no effect on the mutant lipid content or cell envelope integrity, thus raising questions regarding AccD6_*Msm*_ activity and biological function(s)^14^. Assuming that there was no convincing *in vivo* data confirming AccD6_*Msm*_ as the subunit of the active malonyl-CoA-producing enzyme in *Msm,* we sought to address whether AccD6_*Msm*_ and AccD6_*Mtb*_ share the same catalytic activity.

Therefore, we analyzed the possibility of exchanging the functional copy of *accD6*_*Mtb*_ for *accD6*_*Msm*_ in *Mtb* and studied the phenotypic consequences with respect to fatty/mycolic acid biosynthesis. We previously reported the construction of a *Mtb* Δ*accD6*_*Mtb*_-*attB::*P_*fasII*_-*D6*_*Mtb*_ mutant devoid of a chromosomal *accD6*_*Mtb*_ copy and complemented with the same gene under FAS II operon promoter in the integrative pMV306 construct^14^. The expression level of the integrated *accD6*_*Mtb*_ in Δ*accD6*_*Mtb*_-*attB::*P_*fasII*_-*D6*_*Mtb*_ was the same as that for wild-type *Mtb*. Moreover, the integrated *accD6*_*Mtb*_ can be easily replaced by another gene with the same function, provided by an integrative vector that makes the Δ*accD6*_*Mtb*_-*attB::*P_*fasII*_-*D6*_*Mtb*_ strain an ideal tool for testing the activity of other acetyltransferase candidates. Briefly, for heterologous expression of AccD6_*Msm*_ in *Mtb,* the pFD6Ms vector was constructed (see Table S1), which carried the *accD6*_*Msm*_ gene under the control of *Mtb* FAS II operon promoter (P_*fasII*_) to maintain the same expression level as that of the native acetyl-CoA carboxyltransferase gene (Fig. S1A). pFD6Ms was subsequently introduced into the Δ*accD6*_*Mtb*_-*attB::*P_*fasII*_-*D6*_*Mtb*_ strain, and after selection, clones in which the replacement of *accD6*_*Mtb*_ by *accD6*_*Msm*_ occurred were confirmed by Southern blot analysis (Fig. S1B). Selection of *Mtb* clones expressing *accD6*_*Msm*_ instead of *accD6*_*Mtb*_, designated Δ*accD6*_*Mtb*_-*attB::*P_*fasII*_-*D6*_*Msm*_, clearly proves that AccD6_*Msm*_ exhibits overlapping functions with AccD6_*Mtb*_ in this *Mycobacterium*.

To further confirm that AccD6_*Msm*_ acts as an acetyl-CoA carboxyltransferase, the phenotype of Δ*accD6*_*Mtb*_-*attB::*P_*fasII*_-*D6*_*Msm*_ was analyzed. The mutant and the wild-type *Mtb* strains showed similar growth rates and viability (Fig. 2A). Because changes in mycobacterial colony morphology are usually the first sign of abnormal cell lipid metabolism and altered envelope^18^,^19^,^20^,^21^, microscopic examination of the Δ*accD6*_*Mtb*_-*attB::*P_*fasII*_-*D6*_*Msm*_ morphotype was conducted but failed to reveal major changes relative to the wild-type strain (Fig. S2A).

Undisturbed acetyl-CoA carboxyltransferase activity was further confirmed by the TLC analysis of [^14^C]acetate-labeled fatty acid methyl esters (FAMEs) and mycolic acid methyl esters (MAMEs) extracted from Δ*accD6*_*Mtb*_-*attB::*P_*fasII*_-*D6*_*Msm*_ mutant (Fig. 2B). Expression of *accD6*_*Msm*_ instead of *accD6*_*Mtb*_ did not affect the synthesis of fatty acids or each of the three species of mycolic acid in the mutant strain relative to wild-type tubercle bacillus.

The results demonstrate that, despite being nonessential, AccD6_*Msm*_ is a functional acetyl-CoA carboxyltransferase supporting the synthesis of malonyl-CoA.

Because both mutant and wild-type mycolic acids migrated with the same *R*_*f*_ value, it can be assumed that the length of α-, methoxy- and keto- chains remained unchanged. Thus, it should be noted that the two AccD6 acetyltransferases (MSMEG_4329 and Rv2247) have comparable mode of action in *Mtb* host. Although the short-chain fatty acids migrated with the same *R*_*f*_ value in both cases, a difference in abundance could be observed.

Malonyl-CoA synthesis is an essential step in lipid metabolism^6^. Because AccD6_*Msm*_ is dispensable and its deletion does not alter the *Msm* FAS I/FAS II pathway^14^, it can be inferred that another carboxyltransferase(s) must catalyze the transfer of a carboxyl group on acetyl-CoA in this bacterium. We thus investigated which of the other remaining AccD proteins are involved in malonyl-CoA synthesis.

### MSMEG_4717 (AccD1), MSMEG_5492 (AccD2) and MSMEG_5642 (AccD3) proteins are not essential for fatty acid synthesis

We previously assessed the essentiality of *accD1* – *accD6* in *Msm* by directed mutagenesis and found that only MSMEG_6391 and MSMEG_1813 (*Mtb accD4* and *accD5* homologs, respectively) are essential for survival^14^. The TLC/autoradiography analysis of [^14^C]acetate-labeled FAMEs and MAMEs extracted from the ∆*accD1*_*Msm*_, ∆*accD2*_*Msm*_ and ∆*accD3*_*Msm*_ mutants indicated undisturbed synthesis of fatty and mycolic acids (Fig. 3). To test if any of three genes/proteins become essential for mycolic acid biosynthesis in *Msm* Δ*accD6* mutant, double mutants lacking each tested subunit along with AccD6_*Msm*_ (∆*D6*;∆*D1*, ∆*D6*;∆*D2*, ∆*D6*;∆*D3*) were constructed and confirmed by Southern blot analysis (Fig. S3A–C). Again, the TLC-based lipid analysis of the resulting strains did not reveal major differences in the overall fatty acid biosynthesis (Fig. 3), suggesting that AccD1_*Msm*_, AccD2_*Msm*_ and AccD3_*Msm*_ are not essential for FAS I/FAS II pathway.

### MSMEG_6391 (AccD4) carboxylates long-chain substrates only, enabling mycolic acid maturation

**Figure 1:**
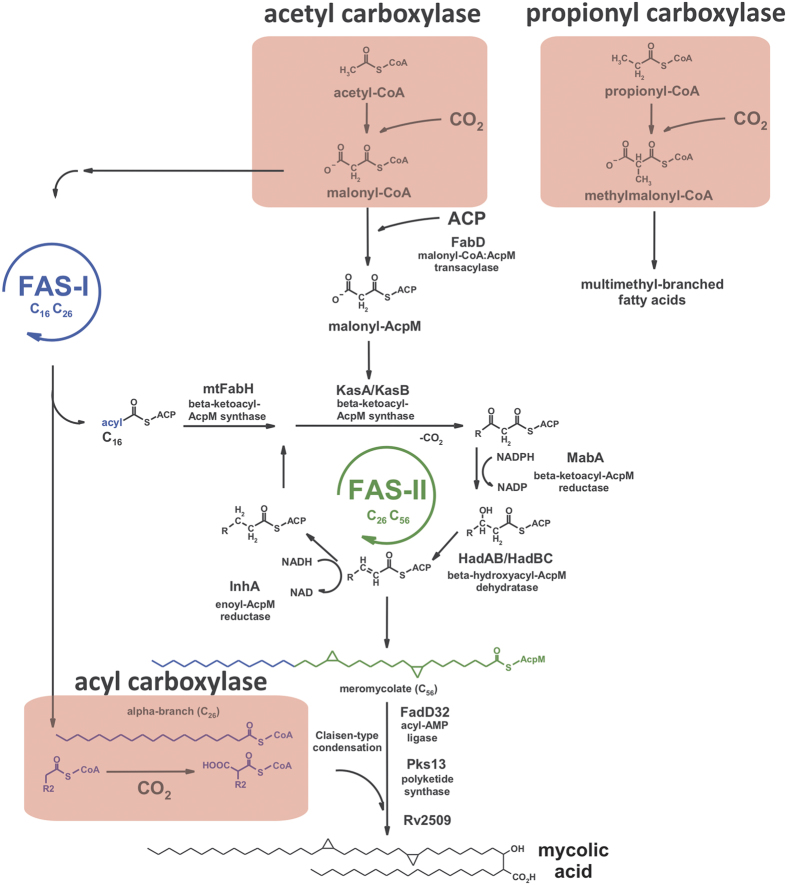
Fatty acid/mycolic acid biosynthesis in mycobacteria. FAS I catalyzes the *de novo* synthesis of short fatty acyl primers C_16_ – C_26_. The C_16_ acyl-CoA product acts as a substrate for the synthesis of meromycolic acid by FAS II, whereas the C_26_ fatty acyl constitutes the α-branch of the mature mycolic acid. FAS II comprises four enzymatic steps catalyzing, via a cyclic reaction, the elongation of short-chain acyl primers to full-length meromycolate chains. The relative contributions of FAS I and FAS II activity in fatty acid/mycolic acid synthesis are represented in blue and green, respectively. The activated α-branch is subsequently condensated by Pks13 with meromycolate converted previously to meromycolyl-AMP. After final reduction, the synthesis of mature mycolic acid is completed. The three stages that require acyl carboxylase activity in fatty/mycolic acid biosynthesis are indicated by red rectangles.

The above-described results suggested that the search for an alternative acetyl-CoA carboxyltransferase in *Msm* should be focused on two essential acyl carboxylase subunits in this bacterium. Despite phylogenetic studies showing that the presence of AccD4 strongly correlates with the ability of the bacterial cell to synthesize mycolic acids^16^, information regarding the substrate specificity of this carboxyltransferase is very limited. The *in vitro* study on purified AccA3 (Rv3285) and AccD4 (Rv3799c) proteins of *Mtb* suggested that AccD4 was unable to transfer the carboxyl group on acetyl/propionyl-CoA but efficiently carboxylated long-chain (C_24_-CoA) substrates during mycolic acid maturation^17^. The genomic organization of *accD4* in mycobacteria supports this thesis because it is found clustered in the same orientation with *pks13* and *fadD32,* genes involved in the final step of mycolic acid synthesis (Fig. 1)^22^. However, the ability of mycobacterial AccD4 to carboxylate long-chain acyl or any other substrate has not yet been confirmed *in vivo*.

This finding prompted us to explore the phenotypes of mycobacteria following AccD4 depletion in order to confirm the previous *in vitro* study^17^. Homologous recombination was used to generate a merodiploid strain in which the MSMEG_6391 (*accD4*_*Msm*_) gene was disrupted in a strain containing another copy of *accD4*_*Msm*_ under the acetamidase inducible promoter (Fig. S3D). To reliably analyze the effect of reduced *accD4*_*Msm*_ expression on malonyl-CoA synthesis, the experiment was carried out in a strain lacking the *accD6*_*Msm*_ gene, thus eliminating the known carboxyltransferase capable of synthesizing this component. The genotype of the constructed mutant, named Δ*D6*;Δ*D4*-*attB::*P_*ami*_-*D4*_*Msm*_, was confirmed by PCR and Southern blot hybridization (Fig. S3D).

To analyze the phenotypic consequences of the AccD4 depletion in *Msm*, fresh cultures of Δ*D6*;Δ*D4*-*attB::*P_*ami*_-*D4*_*Msm*_ were grown in liquid medium supplemented with 0.2% acetamide and centrifuged at an optical density of 0.6. The cell suspension was washed and used for inoculation of the fresh medium, which was or was not supplemented with acetamide. OD measurements revealed normal growth kinetics for Δ*D6*;Δ*D4*-*attB::*P_*ami*_-*D4*_*Msm*_ cells growing in acetamide-containing medium. In contrast, the mutant showed a significant decrease in growth rate, starting at 12 h of growth in the absence of acetamide (Fig. 4A). As indicated by CFU counts, the viability of Δ*D6*;Δ*D4*-*attB::*P_*ami*_-*D4*_*Msm*_ decreased dramatically after 3 h of culture in medium in the absence of acetamide (Fig. 4A). After 24 h, clearing of the growth medium was accompanied by an accumulation of debris, indicating cell lysis (Fig. 4B). In addition, the Δ*D6*;Δ*D4*-*attB::*P_*ami*_-*D4*_*Msm*_ strain was unable to grow on solid medium without acetamide (Fig. 4B). The viability defect observed under non-permissive growth conditions was correlated with the reduced level of *accD4*_*Msm*_ expression, as demonstrated by qRT-PCR (Fig. 4C).

We next investigated the impact of the AccD4 depletion on fatty/mycolic acid biosynthesis, with the levels of both lipids monitored at different time intervals following growth with or without acetamide. Bacterial culture aliquots were collected and pulsed with [^14^C]acetate. FAMEs and MAMEs were extracted and analyzed by TLC/autoradiography. The Δ*D6*;Δ*D4*-*attB::*P_*ami*_-*D4*_*Msm*_ mutant demonstrated a pronounced decrease in the synthesis of three mycolic acid subspecies (α, α′ and epoxy) in the absence of an inducer (Fig. 4C). In contrast, the synthesis of fatty acids was not affected, and their accumulation during the experiment was observed (Fig. 4C). These changes were not observed following lipid extraction of Δ*D6*;Δ*D4*-*attB::*P_*ami*_-*D4*_*Msm*_ grown with acetamide.

There are two stages during straight-chain fatty and mycolic acid synthesis that require the activity of carboxyltransferase: (i) malonyl-CoA synthesis, which involves a two-carbon elongating unit of the aliphatic chain (acetyl-CoA carboxyltransferase); and (ii) activation of the α-branch preceding its condensation with meromycolyl-AMP during mycolic acid maturation (long-chain acyl-CoA carboxyltransferase) (Fig. 1). The lipid analysis of the Δ*D6*;Δ*D4*-*attB::*P_*ami*_-*D4*_*Msm*_ mutant grown without acetamide demonstrated that the lack of AccD6 with a concomitant depletion of AccD4 did not affect the malonyl-CoA synthesis because the short-chain (C_16–24_) fatty acids were still produced. However, the accompanying decline in all species of mature mycolic acid content strongly suggests a lack of long-chain acyl-CoA carboxyltransferase activity. If the α-branch cannot be activated by carboxylation, its condensation with meromycolyl-AMP is blocked, thus leading to the cessation of full-length mycolic acid biosynthesis. Consequently, the fatty acyl primers – the products of FAS I – become unable to enter the final FAS II mycolic acid synthetic step and accumulate (together with yet unidentified intermediates) as indicated by TLC/autoradiography (Fig. 4C).

Overall, our lipid analysis demonstrates that AccD4_*Msm*_ is unlikely the malonyl-CoA-producing carboxyltransferase that functionally compensates for the loss of AccD6_*Msm*_ in the ∆MSMEG_4329 mutant. However, the study confirms *in vivo*, that AccD4 is the carboxyltransferase subunit of the enzyme capable of long-chain acyl-CoA carboxylation in mycobacteria.

To confirm that AccD4 is unable to carboxylate the short-chain substrates, we verified the possibility of swapping *accD4*_*Msm*_ and *accD6*_*Mtb*_ in *Mtb*. Again, for this purpose, replacement of the *attB::*P_*fasII*_-*D6*_*Mtb*_ complementation in the Δ*accD6*_*Mtb*_-*attB::*P_*fasII*_-*D6*_*Mtb*_ mutant was used. The integrative vector carrying *accD4*_*Msm*_ under the control of *Mtb* FAS II promoter (P_*fasII*_) was constructed (see Table S1) and introduced into the *attB* site of the Δ*accD6*_*Mtb*_-*attB::*P_*fasII*_-*D6*_*Mtb*_ mutant. After subsequent selection, the transformants in which *accD6*_*Mtb*_ was potentially replaced by *accD4*_*Msm*_ were subjected to genotype verification. Southern blot analysis revealed the absence of the signal specific to *accD4*_*Msm*_ in the chromosome of the selected mutants, demonstrating that the genetic exchange was not possible (Fig. S2B), despite several attempts. One can therefore exclude the possibility that AccD4_*Msm*_ carboxylates short-chain acetyl-CoA. Altogether, these results strongly emphasize long-chain acyl-CoA as the only substrate of AccD4_*Msm*_ *in vivo*.

### AccD5 of *M. smegmatis* (MSMEG_1813) but not that of *M. tuberculosis* (Rv3280) acts as acetyl-CoA carboxyltransferase *in vivo*

In view of the above-mentioned results, the protein encoded by the MSMEG_1813 gene, homologous to *Mtb accD5* (Rv3280), became the last possible candidate as an alternative acetyl-CoA carboxyltransferase in *Msm*. The substrate specificity of AccD5 was deduced from comparative studies on the homologous carboxylase subunit of *C. glutamicum* (Cg0811) and *S. coelicolor* (SCO4926)^12^,^18^,^23^. Despite clear substrate preference for propionyl-CoA, the enzyme also presented a minor ability to bind and carboxylate acetyl-CoA. Such bi-substrate specificity was also reported *in vitro* on the *Mtb* carboxylase reconstituted from AccA3 and AccD5 protein^16^,^17^. As demonstrated on *S. coelicolor* PccB (SCO4926), the ability to bind both acetyl- and propionyl-CoA may be determined by the presence of a single cysteine (Cys422) in the enzyme active site pocket^16^. Sequence analysis of *Mtb* AccD5 revealed a conserved cysteine residue in the corresponding position (Cys437), suggesting common substrate specificity. However the exact function and ability to utilize any of the mentioned substrates have not been confirmed *in vivo*.

The homology analysis revealed that MSMEG_1813 (AccD5_*Msm*_) protein shares 88% sequence identity with AccD5_*Mtb*_ (Rv3280) and confirmed strong conservation of the regions determining the secondary structure and the overall fold (Fig. S4). The alignment of the known structure of AccD5_*Mtb*_ monomer with the deduced *in silico* structure of AccD5_*Msm*_ indicated the obvious structural homology that extends to key motifs responsible for biotin binding (Gly434; Ala435), substrate binding (Gly193–194) or the ability to bind both acetyl- and propionyl-CoA (Cys437) in the active site pocket (Fig. S5). Thus, by analogy with AccD5_*Mtb*_, the bi-substrate specificity of AccD5_*Msm*_ protein is also possible and may allow explaining its functional overlap with AccD6_*Msm*_. This prompted us to investigate *in vivo* the possible ability of AccD5_*Msm*_ to carboxylate acetyl-CoA.

As reported above, AccD6_*Msm*_ functionally replaced the essential acetyl-CoA carboxylase of *Mtb* (AccD6_*Mtb*_). We next addressed whether such replacement is also possible with AccD5_*Msm*_. Again, to test the ability of AccD5_*Msm*_ to support the acetyl-CoA carboxyltransferase function in tubercle bacillus, the *Mtb* Δ*accD6*_*Mtb*_-*attB::*P_*fasII*_-*D6*_*Mtb*_ mutant was applied. The *accD5*_*Msm*_ gene was cloned under the control of *Mtb* P_*fasII*_ promoter into a pMV306 vector. The construct carrying *accD5*_*Msm*_, named pFD5Ms (see Table S1), was subsequently introduced into the Δ*accD6*_*Mtb*_-*attB::*P_*fasII*_-*D6*_*Mtb*_ strain (Fig. S6A). After selection, clones in which the potential exchange of functional *accD6*_*Mtb*_ for *accD5*_*Msm*_ occurred were subjected to Southern blot genotype analyses (Fig. S6B). The presence of the expected hybridization signal confirmed the successful exchange; the resulting mutant strain was named Δ*accD6*_*Mtb*_-*attB::*P_*fasII*_-*D5*_*Msm*_. At the same time, the absence of the signal in negative controls confirmed that the *accD5*_*Msm*_ probe shows no tendency for non-specific binding to the *accD6*_*Mtb*_ gene or any other sequence of the *Mtb* chromosome. The results demonstrated that *accD5*_*Msm*_ is able to replace the essential acetyl-CoA carboxyltransferase gene in *Mtb*.

Therefore, it can be assumed that AccD5_*Msm*_ supports the physiological level of malonyl-CoA in tubercle bacillus. To demonstrate that the synthesis of the essential fatty acid building block malonyl-CoA was not disturbed in the mutant strain, we analyzed its growth parameters, colony morphology and, most importantly, the level of fatty acid biosynthesis.

The growth rate analysis revealed a slight difference in optical density between cultures of wild-type *Mtb* and the Δ*accD6*_*Mtb*_-*attB::*P_*fasII*_-*D5*_*Msm*_ mutant, starting from 96 h of incubation; however, as indicated by CFU counts, the viability of the two strains were comparable at the end of the experiment (Fig. 5). The colony morphology of the *Mtb* Δ*accD6*_*Mtb*_-*attB::*P_*fasII*_-*D5*_*Msm*_ mutant did not differ from that of the wild-type strain (Fig. S2A), suggesting the absence of any severe alterations in the mutant cell envelope lipids. However, the colonies monitored over time grew noticeably smaller in diameter than the wild-type *Mtb* colonies.

**Figure 2:**
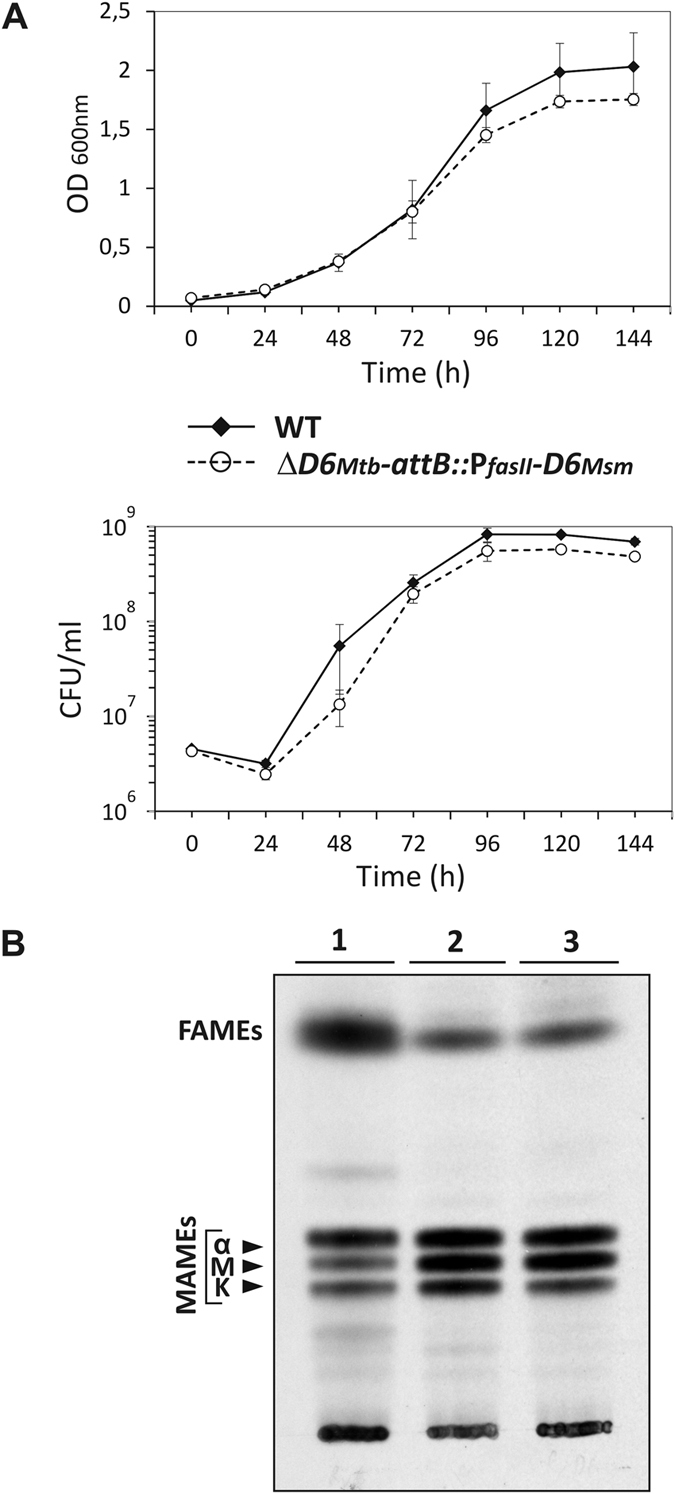
(**A**) The growth of Δ*accD6*_*Mtb*_-*attB::*P_*fasII*_-*D6*_*Msm*_ mutant (dashed line) in relation to *Mtb* wild-type (solid line) was followed by measuring the OD_600 nm_ (top) and the number of viable cells (bottom). Values are means ± standard errors from three independent experiments. (**B)** Thin-layer chromatography of ^14^C-labeled fatty acid methyl esters (FAMEs) and mycolic acid methyl esters (MAMEs) extracted from the cells of **1** - *Mtb* wild-type strain, **2** - Δ*accD6*_*Mtb*_-*attB::*P_*fasII*_-*D6*_*Msm*_ mutant, **3** - Δ*accD6*_*Mtb*_-*attB::*P_*fasII*_-*D5*_*Msm*_ mutant. Equal counts (15 000 cpm) were loaded onto TLC plate and developed in hexane – ethyl acetate (19:1, vol/vol). The symbols α, M, and K correspond to α-mycolates, methoxy-mycolates and keto-mycolates, respectively.

**Figure 3:**
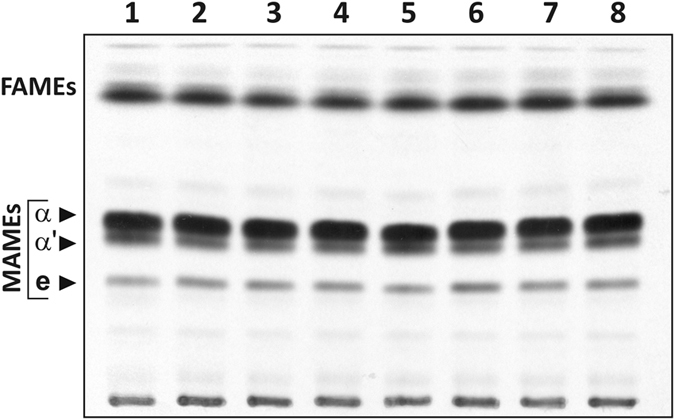
Analysis of *Msm* knockout mutants fatty and mycolic acid biosynthesis. Thin-layer chromatography of ^14^C-labeled fatty acid methyl esters (FAMEs) and mycolic acid methyl esters (MAMEs) extracted from the cells of **lines 1** – *Msm* wild-type strain, **2** - ∆*accD1* mutant, **3** - ∆*accD2* mutant, **4** - ∆*accD3* mutant, **5** - ∆*accD6* mutant, **6** - ∆*D6*;∆*D1* mutant, **7** - ∆*D6*;∆*D2* mutant, **8** - ∆*D6*;∆*D3* mutant. Equal counts (100 000 cpm) were loaded onto TLC plate and developed in hexane – ethyl acetate (95:5, vol/vol). The symbols α, α′, and e correspond to α-mycolates, α’-mycolates and epoxy-mycolates, respectively.

**Figure 4:**
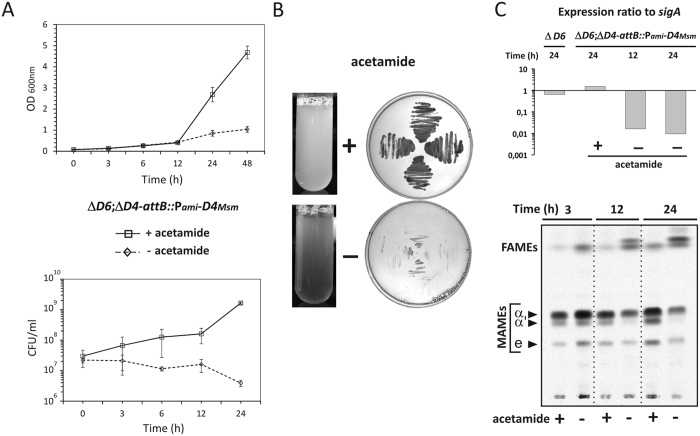
Analysis of the Δ*D6*;Δ*D4*-*attB::*P_*ami*_-*D4*_*Msm*_ conditional mutant phenotype. (**A**) Growth in 7H9/OADC medium with (solid line) and without (dashed line) acetamide was followed over time by measuring the OD_600 nm_ (top) and number of viable cells (bottom). Values are means ± standard errors from three independent experiments. (**B)** (left) Images of the mutant liquid cultures after 24 h of growth with (+) and without (−) acetamide. (right) Mutant growth on 7H10/OADC agar with (+) and without (−) acetamide. (**C)** (top) Monitoring of the *accD4*_*Msm*_ expression level. The *accD4*_*Msm*_ transcript level was measured in mutant strain growing for 12 and 24 h in 7H9/OADC medium with (+) and without (−) acetamide. The expression level of the same transcript was also measured in the ∆*accD6* parental strain after 24 h of culture. (bottom) Thin-layer chromatography of ^14^C-labeled fatty acid methyl esters (FAMEs) and mycolic acid methyl esters (MAMEs) extracted from the mutant after 3, 12 and 24 h of culture in 7H9/OADC medium with (+) and without (−) acetamide. Equal counts (10 000 cpm) were loaded onto TLC plate and developed in hexane – ethyl acetate (19:1, vol/vol). The symbols: α, α′, and e correspond to α-mycolates, α′-mycolates and epoxy-mycolates, respectively.

**Figure 5:**
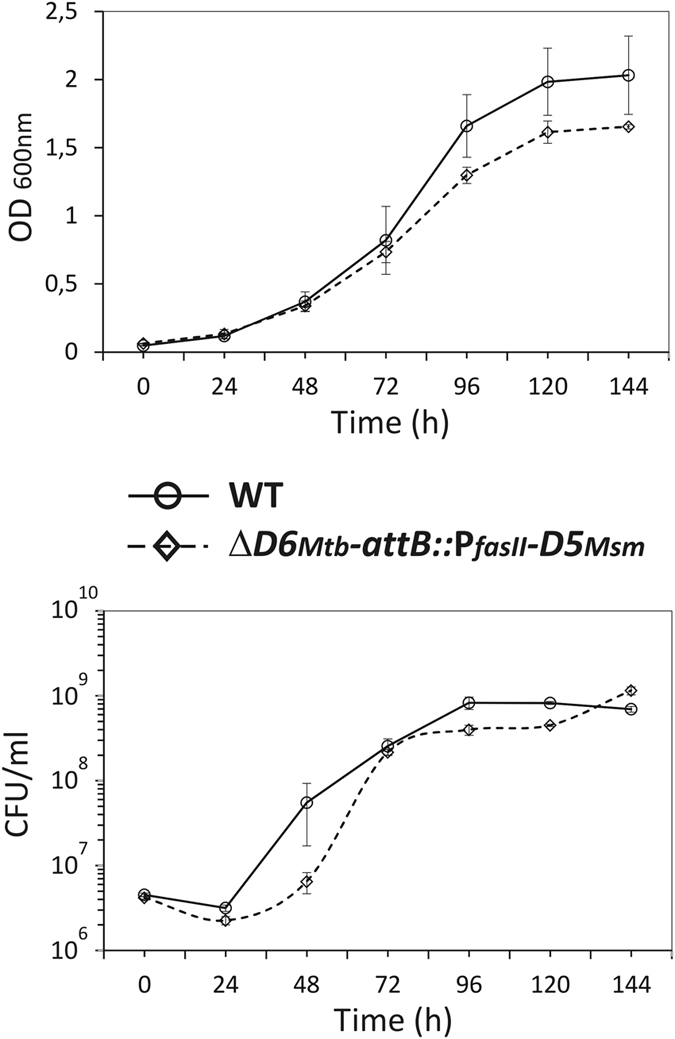
The growth of Δ*accD6*_*Mtb*_-*attB::*P_*fasII*_-*D5*_*Msm*_ mutant (dashed line) in relation to *Mtb* wild-type (solid line) was followed by measuring OD_600 nm_ (top) and the number of viable cells (bottom). Values are means ± standard errors from three independent experiments.

TLC analysis of the [^14^C]acetate-labeled FAMEs and MAMEs ultimately confirmed that AccD5_*Msm*_ effectively replaces the function of the AccD6_*Mtb*_ acetyl-CoA carboxyltransferase in tubercle bacillus because fatty acids and all species of mycolic acids characteristic for *Mtb* were produced in the Δ*accD6*_*Mtb*_-*attB::*P_*fasII*_-*D5*_*Msm*_ strain. No additional radioactive lipid intermediates could be observed (Fig. 2B).

This observation led us to the conclusion that inactivation of the functional carboxyltransferase subunit of the malonyl-CoA-producing enzyme in *Msm* – MSMEG_4329 (AccD6_*Msm*_) – was possible because of an additional protein sharing the same substrate specificity - MSMEG_1813 (AccD5_*Msm*_). To the best of our knowledge, this study is the first to confirm the *in vivo* ability of AccD5 to carboxylate acetyl-CoA in *Msm*.

The study of the malonyl-CoA synthesis pathway in fast-growing *Msm* prompted us to conclude that the inability to inactivate *accD6* (Rv2247) in *Mtb* may be directly related to *in vivo* catalytic characteristics of AccD5 (Rv3280) in this pathogen. Despite high sequence and structure homology between AccD5 of *Mtb* and that of *Msm*, the failure to obtain the ∆*accD6* mutant in tubercle bacillus strongly suggests that AccD5_*Mtb*_ is unable to exhibit its acetyl-CoA carboxylase activity *in vivo*. However, based on existing *in vitro* studies indicating the bi-substrate (acetyl/propionyl) specificity of AccD5_*Mtb*_, we next addressed whether the inability of this protein to replace the function of AccD6_*Mtb*_ may be related to lower (compared with that observed for *Msm*) acetyl-CoA carboxyltransferase activity, which may also be accompanied by a higher requirements for malonyl-CoA in this pathogen. Thus, we analyzed whether an increase in the *accD5*_*Mtb*_ copy number in *Mtb* cells makes *accD6*_*Mtb*_ non-essential.

To compare the results obtained from the parallel study on the AccD5 of the pathogenic and fast-growing mycobacterial strain, we used the same vector and host implemented in the construction of the Δ*accD6*_*Mtb*_-*attB::*P_*fasII*_-*D5*_*Msm*_ mutant to introduce an extra copy of *accD5*_*Mtb*_ into tubercle bacillus cells. To express AccD5_*Mtb*_ at the same level as AccD6_*Mtb*_, the *accD5*_*Mtb*_ gene was cloned under the control of *Mtb* FAS II operon promoter (P_*fasII*_) into a pMV306 vector. The resulting construct, named pFD5Tb (see Table S1), was used to replace the pMV306 vector carrying P_*fasII*_-*D6*_*Mtb*_ in the Δ*accD6*_*Mtb*_-*attB::*P_*fasII*_-*D6*_*Mtb*_ strain. The selection of *Mtb* transformants without an intact *accD6* carrying an additional copy of *accD5*_*Mtb*_ under control of P_*fasII*_ was not successful, suggesting that unlike AccD5_*Msm*_, the AccD5_*Mtb*_ protein is not able to functionally replace the essential AccD6_*Mtb*_ (Rv2247), even after increasing the *accD5*_*Mtb*_ gene copy number (Fig. S2C).

## Discussion

“The substrate specificity of AccD1–6 has been an unsolved mystery…^24^”.

Extensive *in vitro* studies have allowed for the proposal of the possible catalytic activity of the three essential carboxyltransferase subunits in tubercle bacillus: AccD4 (Rv3799c), AccD5 (Rv3280) and AccD6 (Rv2247)^15^,^16^,^17^. However, these *in vitro* data were poorly assessed in mycobacterial cell. The *in vivo* confirmation of mycobacterial acyl carboxylase substrate specificity appears to be the most vital in obtaining a full picture of the process that leads to the synthesis of full-length, mature mycolates as well as other straight- and branched-chain fatty acids. We previously provided the first *in vivo* evidence confirming that *accD6* (Rv2247) is an essential gene encoding the functional acetyl-CoA carboxylase subunit in *Mtb*, and, unexpectedly, we reported the non-essential character of the *M. smegmatis* homolog MSMEG_4329 (*accD6*_*Msm*_)^14^, although it has been described by others as an essential gene^25^. In our study, the lipid profile of the ∆*accD6*_*Msm*_ mutant was not affected, emphasizing the participation of an AccD6–independent acetyl-CoA carboxylase in *Msm*^14^.

Herein, we dissected the relationship and potential role in fatty acid biosynthesis of the six AccD carboxyltransferases *in vivo*, with the goal of identifying the candidate capable of carboxylating acetyl-CoA and to reveal the direct cause of the difference in *accD6* essentiality between pathogen and fast-growing species. Because the *in vivo* function of AccD6 in *Mtb* was previously confirmed, we reasoned that a suitable way to verify the *in vivo* acetyl-CoA carboxyltransferase activity of the various AccDs would rely on testing the possibility of gene exchange between *accD6*_*Mtb*_ and other carboxyltransferase encoding genes. For this purpose, the *Mtb* Δ*accD6*_*Mtb*_-*attB::*P_*fasII*_-*D6*_*Mtb*_ strain was used as a recipient strain because the only functional copy of *accD6* in this strain is localized on an integrated plasmid and can be easily exchanged in one site-specific recombination step by other gene supporting its function. Our phenotypic analysis established that *accD6*_*Mtb*_ and *accD6*_*Msm*_ encode active proteins of convergent catalytic activity *in vivo* and that the dispensability of AccD6_*Msm*_ suggested the presence, in *Msm,* of another carboxyltransferase sharing overlapping activity to sustain the synthesis of malonyl-CoA. We screened all five remaining *Msm* carboxyltransferase genes – *accD1*_*Msm*_ to *accD5*_*Msm*_ – in terms of their involvement in mycolic acid biosynthesis and ability to use acetyl-CoA as the substrate for carboxylation. As previously reported, among all six *accD* homologs in *Msm*, only MSMEG_6391 (*accD4*_*Msm*_) and MSMEG_1813 (*accD5*_*Msm*_) are essential for bacterial survival^14^. When testing *accD1*_*Msm*_, *accD2*_*Msm*_ and *accD3*_*Msm*_, disrupted either individually or together with *accD6*_*Msm*_ to eliminate the residual acetyl-CoA carboxyltransferase activity of AccD6, all mutants showed a lipid profile comparable with that of the wild-type strain, thus ruling out the essential function of AccD1 – AccD3 in the fatty acid synthesis in *Msm*. As reported by another group, the simultaneous deletion of *accD1*/*accA1* or *accD2*/*accA2* also failed to induce changes in the lipid metabolism of *Msm*, thus eliminating AccA1 and AccA2 proteins as biotin carboxylase subunits involved in fatty/mycolic acid biosynthesis, leading the authors to propose the involvement of the AccD1-AccA1 complex in branched amino-acid catabolism with methylcrotonyl-CoA as the substrate^26^. The metabolic function of the AccD2 and AccD3 carboxyltransferases awaits further study.

The AccD4 protein was the least characterized essential carboxyltransferase in mycobacteria. Its activity has been predicted *in vitro* but has never been confirmed in mycobacteria. Herein, we demonstrated in *Mycobacterium* cell the role of AccD4 in mycolic acid synthesis as the subunit of long-chain acyl carboxylase, confirming the previous *in vitro* study^17^. To unravel its possible acetyl-CoA carboxylase activity, conditional AccD4_*Msm*_ depletion was carried out in the strain lacking functional *accD6*_*Msm*_. The fatty and mycolic acid biosynthesis pattern following AccD4 depletion indicates that malonyl-CoA was continuously synthesized throughout the entire depletion experiment, allowing the short-chain FAS I products synthesis. Strikingly, the gradual disappearance of all three species of mycolic acid could be observed. The FAMEs/MAMEs TLC profile prior to the mutant cell lysis mimics, to a certain extent, the “mycolate-less” profile of *C. glutamicum* lacking the *accD4* homolog^11^, confirming the loss of the long-chain acyl-CoA carboxylase, activating the α-branch prior to its condensation with meromycolate. Moreover, the ability of *Msm* AccD4 to carboxylate acetyl-CoA was also excluded because *accD4*_*Msm*_ was unable to replace the acetyl-CoA carboxyltransferase-encoding gene in *Mtb*.

AccD5_*Msm*_ was the last candidate analyzed for the alternative subunit of acetyl-CoA carboxylase in *Msm*. Although *in vitro* studies on AccD5_*Mtb*_ showed substrate preference for propionyl-CoA, the enzyme also exhibited modest activity with acetyl-CoA^16^. Since determining its crystal structure^24^, AccD5 has become the best studied mycobacterial carboxyltransferase, although there have been no convincing *in vivo* data confirming any of the activities attributed to this protein. Bazet Lyonnet *et al*., described the phenotype of D5DCO conditional mutant in *Msm*^27^. Because the MSMEG_1813 (*accD5*_*Msm*_) gene is co-transcribed with MSMEG_1812 (*accE5*_*Msm*_), generating the conditional *accD5* mutant without affecting the neighboring *accE5* remains challenging. Also in the case of the D5DCO mutant, the conditional depletion encompassed both AccD5 and AccE5 and resulted in a highly pleiotropic effect on all types of acyl carboxylase activity. Because *accE5* encodes a possible regulatory protein interacting with AccD5 and other carboxyltransferases^17^,^26^, we cannot exclude that the observed D5DCO mutant phenotype was, in fact, the result of the polar effect of AccE5 depletion on the activity of all acyl carboxylase complexes. Because any of two possible activities of AccD5 has never been conclusively confirmed in mycobacteria, we verified the *in vivo* relevance of AccD5 to the carboxylation of acetyl-CoA. Successful replacement of *accD6* in *Mtb* with *accD5*_*Msm*_ strongly supports the acetyl-CoA carboxyltransferase activity of AccD5_*Msm*_ and explains the nonessential nature of *accD6* in *Msm*. Because both AccD5_*Msm*_ and AccD6_*Msm*_ catalyze acetyl-CoA carboxylation, the question arises whether AccD5_*Msm*_ only complements the function of AccD6_*Msm*_ in ∆*accD6*_*Msm*_ mutant or acts as a major subunit of malonyl-CoA-producing carboxylase in *Msm*. Phenotypic characterization of the ∆*accD6*_*Msm*_ mutant demonstrated that despite the absence of any significant changes in lipid metabolism, the AccD6_*Msm*_ deletion affected bacterial growth^14^^,data not shown^. The decrease in the mutant growth rate and viability could be reversed after the delivery of functional *accD6*_*Msm*_ or *accD6*_*Mtb*_ to the mutant. It should also be noted that the *accD6* expression level during intensive mycolic acid biosynthesis is the highest among the carboxyltransferase genes in both *Mtb* and *Msm*^14^,^15^. Therefore, this work again emphasizes the central role of AccD6 as the subunit of acetyl-CoA carboxylase in both pathogenic and free-living mycobacteria. Interestingly, because the *accD6* interchange between the analyzed species has no effect on the reaction catalyzed, it appears that the mechanisms and interactions leading to the formation of α/β subunit dimers or active carboxylase holoenzyme assembly are highly conservative among mycobacteria.

Because *accD6*_*Mtb*_ is required for *Mtb* viability^14^, one can assume that demonstrated in previous *in vitro* experiments^16^ activity of AccD5 (Rv3280) in carboxylation of acetyl-CoA is insufficient for the replacement AccD6_*Mtb*_ function *in vivo*. However, the introduction of the extra copy of *accD5*_*Mtb*_ under P_*fasII*_ promoter was not sufficient to render the *accD6*_*Mtb*_ gene dispensable, suggesting that inability of the AccD5_*Mtb*_ to exhibit *in vivo* the acetyl-CoA carboxyltransferase activity represents more complex problem, requiring further study.

Overall, our findings link the differences in AccD6 essentiality to variations in AccD5 activity in fast- and slow-growing mycobacteria. Despite their high structural conservation, AccD5 proteins from saprophytic *Msm* and pathogenic *Mtb* differ in their substrate preference. Our *in vivo* analysis did not confirm the postulated *in vitro* acetyl/propionyl-CoA bi-substrate specificity of AccD5_*Mtb*_. Because AccD5_*Mtb*_ was unable to exhibit its acetyl-CoA carboxyltransferase activity, it can be suspected that despite the verified *in vitro* biochemical potential of bi-substrate specificity, AccD5 of tubercle bacillus is directed *in vivo* solely to catalyze propionyl-CoA carboxylation.

The difference in AccD5 behaviour between pathogenic and non-pathogenic species may depend on the specific role of propionyl-CoA carboxylase in mycobacterial metabolism. Indeed, propionyl-CoA carboxylase activity can be considered important for *Mtb* pathogenicity. The conversion of propionyl-CoA to methylmalonyl-CoA provides the key chain-extending unit for the synthesis of multimethyl-branched fatty acids (e.g., mycocerosic acid) that constitute potent tubercle bacillus virulence factors, modulating host cell function^9^,^28^,^29^. Munoz-Elias *et al*., postulated that the intensive synthesis of methyl-branched lipids is the major pathway of cytosolic propionyl-CoA metabolism during *Mtb* infection in mice^30^. Propionyl-CoA carboxylase activity is also the first and limiting step of the methylmalonyl-CoA mutase-dependent pathway of propionate metabolism (methylmalonyl pathway). Savvi *et al*., established the capacity of the methylmalonyl pathway to fulfill a key role in propionate metabolism during the growth of *M. tuberculosis* on fatty acids of odd chain length, in some cases being the preferred metabolic route (e.g., growth on valerate)^31^. The methylmalonyl pathway also enables the utilization of propionate as the sole carbon source in the absence of both the glyoxylate and methylcitrate cycles^31^. Because glucose deficiency and an abundance of fatty acids are believed to be typical conditions that *Mtb* meets in the host cell^30^,^32^, the activity of propionyl-CoA carboxylase together with methylmalonyl-CoA mutase may expand the spectrum of catabolized host lipids and increase the efficiency with which excess toxic propionate is removed from the cell.

Given the high demand for propionyl-CoA carboxylase activity in *Mtb* pathogenesis, AccD5 protein in this bacterium may have undergone a strict evolutionary specialization towards the utilization of propionyl-CoA as the sole substrate of the reaction catalyzed. In *Msm*, however, the synthesis of the most representative methyl-branched tuberculostearic acid (10-methylstearic) can be considered “AccD5-independent” because the methyl moiety is derived from the S-methyl group of methionine in this case^33^,^34^. Thus, it can be expected that the requirement for propionyl-CoA carboxylase activity in *Msm* may be considerably lower than that in pathogenic species and that the substrate specificity of AccD5 in this bacterium may evolve towards acetyl-CoA utilization. Because the acetyl-CoA carboxyltransferase activity of AccD5 in this species duplicates the activity of AccD6, AccD5_*Msm*_ essentiality is very likely dependent on its second propionyl-CoA carboxyltransferase function. Indeed, the presence of the active methylmalonyl pathway was also confirmed in *Msm*^35^, but its importance for the saprophyte metabolism awaits further study.

## Methods

### Bacterial strains and culture conditions

*M. tuberculosis* H37Rv (ATCC), *M. smegmatis* mc^2^155 and *E. coli* One Shot TOP10 (Invitrogen) were used in the present study^36^,^37^. Strains based on *M. tuberculosis* and *M. smegmatis* were maintained on Middlebrook 7H10 agar or 7H9 broth (Becton Dickinson) with 10% OADC (oleic acid, albumin, dextrose, catalase) enrichment (Becton Dickinson). For selection, we used kanamycin (25 μg ml^−1^), hygromycin (50 μg ml^−1^), gentamicin (7.5 μg ml^−1^), 5-bromo-4-chloro-3-indolyl-β-D-galactopyranoside (X-Gal; 50 μg ml^−1^), or sucrose (2%, wt/vol) as appropriate. *E. coli* was used as the host for cloning and was grown in LB medium. Plasmid selection and maintenance were performed using ampicillin (10 μg ml^−1^), chloramphenicol (34 μg ml^−1^), hygromycin (200 μg ml^−1^), and kanamycin (50 μg ml^−1^). The plasmids used in this study are listed and described in Table S1. Culture densities were determined using a BioPhotometer plus (Eppendorf); the results presented reflect the values remaining after subtracting the optical density at 600 nm (OD_600_) of the culture medium. To determine the numbers of CFU, aliquots of cultures were serially diluted in 7H9/OADC (10-fold dilutions). The appropriate dilutions were then spread on 7H10/OADC agar, and the plates were incubated at 37 °C before CFU were counted. The bacterial colony morphology was studied using a Nikon SMZ1500 Stereoscope (objective: HR Plan Apo 1x WD54, ocular: C-W 10x A/22).

### Gene cloning strategies

Standard molecular biology protocols were used for all cloning procedures^38^. All PCR products were obtained using thermostable AccuPrime *Pfx* DNA polymerase (Invitrogen). They were initially cloned into a pJET1.2/blunt vector (Thermo Scientific), followed by sequencing and digestion with the appropriate restriction enzymes. They were then cloned into the final vectors. To facilitate subcloning, certain restriction enzyme recognition sites were incorporated into the primer sequences (see Table S2), whereas in other cases, natural restriction sites were used.

### Construction of *M. smegmatis accD1 – accD4* gene replacement vectors

To create an unmarked deletion of (MSMEG_4717) *accD1*, (MSMEG_5492) *accD2*, (MSMEG_5642) *accD3* and (MSMEG_6391) *accD4* for each gene, a suicidal recombination delivery vector based on p2NIL was used^39^. Each vector carried the region upstream of *accD* gene together with the 5′ end of the gene (GR1-GR2) cloned next to the 3′ end of the gene and its downstream region (GR3-GR4). The GR1-GR2 and GR3-GR4 fragment lengths as well as the PCR primers used for their amplification on *M. smegmatis* chromosomal DNA are listed in Table S2. The GR1-GR2 and GR3-GR4 PCR fragments of each gene were ligated into p2NIL such that the reconstituted ∆*accD1*, ∆*accD2*, ∆*accD3*, and ∆*accD4* genes were devoid of an internal sequence (*accD1* – 938 bp, *accD2* – 981 bp, *accD3* – 822 bp, *accD4* – 966 bp). Because all genes were cloned out of frame, they encoded nonfunctional proteins. Finally, the PacI screening cassette from pGOAL17 was inserted into the prepared constructs^39^, yielding the suicide delivery vectors: pJPD1Ms, pJPD2Ms, pJPD3Ms, and pJPD4Ms (Table S1).

### Disruption of the *M. smegmatis accD1 – accD3* genes by homologous recombination

The two-step homologous recombination protocol of Parish and Stoker^39^ was used to introduce unmarked deletions into the *accD1, accD2* and *accD3* genes of *M. smegmatis*. The plasmid DNA of pJPD1Ms, pJPD2Ms, and pJPD3Ms suicide delivery vectors was UV-treated (100 mJ) and electroporated into wild-type *M. smegmatis* or ∆*accD6*_*Msm*_ mutant competent cells, where it was integrated into the chromosome by homologous recombination. The resulting single-crossover (SCO) recombinant mutant colonies were blue, kanamycin-resistant, and sensitive to sucrose (2%). The recombination site was confirmed by PCR and Southern blot hybridization. For each gene, a single SCO colony was then selected, resuspended in fresh 7H9 medium with OADC, poured onto 7H10/OADC agar without any selective markers, and incubated at 37 °C for 2 days to allow the second crossover to occur. Serial dilutions were plated onto medium containing sucrose and X-Gal to select for double crossovers (DCO). Potential double-crossover colonies (white, sucrose resistant) carrying either wild-type or the mutated gene variant were screened for kanamycin sensitivity and confirmed by PCR and Southern blot hybridization. Southern blot analysis to distinguish between SCO, wild-type DCO and mutated DCO was performed on a chromosomal DNA template digested with PvuII/SmaI (*accD1*), NotI (*accD2*), and SmaI (*accD3*). The *accD1, accD2* and *accD3* hybridization probes were generated by PCR on pJPD1Ms, pJPD2Ms, pJPD3Ms plasmid DNA as the template, using MsD1co-s/MsD1co-r, MsD2co-s/MsD2co-r and MsD3co-s/MsD3co-r primer pairs, respectively. The same primers were used for PCR genotype confirmation. Probe labeling, hybridization, and signal detection were performed using the AlkPhos Direct labeling and detection system (GE Healthcare) according to the manufacturer’s instructions.

### Construction of ΔD6;ΔD4-attB::P_ami_-D4_Msm_ conditional mutant

For conditional depletion of AccD4_*Msm*_ in the ∆*accD6*_*Msm*_ mutant, the above-described suicide delivery vector - pJPD4Ms - was UV-treated and electroporated into *M. smegmatis* ∆*accD6* mutant competent cells, where it was integrated into the chromosome by homologous recombination. The disruption of MSMEG_4329 (*accD6*) gene in *M. smegmatis* was described previously^14^. The resulting single-crossover (SCO) recombinant mutant colonies were confirmed by PCR and Southern blot hybridization as described below. To disrupt the native chromosomal copy of *accD4*_*Msm*_, another copy of this gene was introduced into the SCO strain on a pAceD4Ms vector. To construct pAceD4Ms, the 1569 bp PCR fragment carrying the whole MSMEG_6391 (*accD4*) gene was amplified on a *M. smegmatis* chromosomal DNA template, using accD4Xbs and accD4Xbr primers. The PCR-amplified gene was then cloned into the XbaI site of the pJam2 vector and subsequently released together with acetamidase promoter (P_*ami*_) by HindIII/BamHI digestion. The resulting fragment was finally ligated into pMV306Hyg integrative vector to yield pAceD4Ms. After integration of the pAceD4Ms into the chromosome, the pAceD4Ms became the source of the functional *accD4*_*Msm*_ gene in the SCO strain growing in medium containing hygromycin and acetamide. It was therefore possible to select the double crossovers (DCO) in which the chromosomal copy of *accD4*_*Msm*_ was replaced by the same gene under acetamidase promoter, integrated on pAceD4Ms vector. White kanamycin-susceptible DCO colonies able to grow on sucrose and hygromycin were then tested for their growth on acetamide. The genotype of selected DCO clones that were able to grow only on acetamide-containing medium were verified by PCR, using the accD4Xbs primer complementary to the 5′ end of *accD4*_*Msm*_ and the MSaccD4GR4 primer complementary to the 3′ end of the GR3–GR4 gene flanking sequence. Finally, the genotype of the Δ*D6*;Δ*D4*-*attB::*P_*ami*_-*D4*_*Msm*_ mutant was confirmed by Southern blot hybridization on the chromosomal DNA template, digested with KpnI, PstI and XbaI restriction enzymes. The *accD4*_*Msm*_ probe was generated by PCR on a pJPD4Ms vector plasmid DNA template, using MsD4co-s and MsD4co-r primers. Probe labeling, hybridization, and signal detection were performed as described above.

### Conditional depletion of AccD4_Msm_

The Δ*D6*;Δ*D4*-*attB::*P_*ami*_-*D4*_*Msm*_ strain was grown in 7H9 medium with OADC containing 0.2% acetamide to an OD_600_ 0.6. The cells were washed three times in the same medium to remove traces of acetamide and used for inoculation of fresh medium to an OD_600_ 0.05. The culture was divided into two aliquots: one with and one without acetamide. To prevent P_*ami*_ promoter leakage, disodium succinate (0.5%) was added to the culture without acetamide. This inoculation corresponds to the 0 h time point for all experiments designed to test cell density and viability and the biochemical effects of AccD4_*Msm*_ depletion. For lipid analysis, the Δ*D6*;Δ*D4*-*attB::*P_*ami*_-*D4*_*Msm*_ mutant culture aliquots were withdrawn at hours 3, 12 and 24 of the depletion experiment and labeled with [^14^C]acetate for 1 h prior to extraction.

### qRT-PCR

For quantitative real-time PCR (qRT-PCR) analysis of *accD4*_*Msm*_ expression, RNA was extracted from wild-type *M. smegmatis* and Δ*D6*;Δ*D4*-*attB::*P_*ami*_-*D4*_*Msm*_ mutant growing with or without acetamide using the TRIzol LS reagent (Invitrogen) as described previously^14^. The SuperScript III First-Strand Synthesis SuperMix (Invitrogen) was used for reverse transcription according to the manufacturer’s instructions. Subsequently, 1 μl of cDNA (equivalent to 50 ng of RNA) was used in the qRT-PCR experiment. qRT-PCR was performed using the Maxima SYBR green qPCR master mix (Thermo Scientific) and a 7900HT real-time PCR system (Applied Biosystems). Each reaction (final volume, 25 μl) was mixed on ice and contained 1 x Maxima SYBR green qPCR master mix, 50 ng of cDNA, and 0.3 μM of each primer (Table S2). The cycling protocol included initial heating to 95 °C for 10 min and 40 cycles of 95 °C for 20 s (denaturation), 63 °C for 30 s (annealing), and 72 °C for 30 s (extension). To verify the specificity and identity of the PCR products generated, a melting curve analysis was performed at the end of each PCR, and the PCR products were analyzed by agarose gel electrophoresis. The results are presented as cycle threshold (*C*_*T*_) values, normalized with respect to the expression of MSMEG_2758 (*sigA*) (∆*C*_*T*_) and converted to a linear form (2^−∆*CT*^).

### *accD6* (Rv2247) gene exchange in *M. tuberculosis*

For *accD6* (Rv2247) gene exchange in *M. tuberculosis*, the pFD6Ms, pFD5Ms, pFD4Ms and pFD5Tb vectors were constructed. Each vector was based on pMV306Km, in which the 1028 bp fragment carrying the *Mtb* FAS II operon promoter (P_*fasII*_) was cloned into NotI/XbaI restriction site to give pMVFAS2 Km. The entire *accD6*_*Msm*_ (MSMEG_4329), *accD5*_*Msm*_ (MSMEG_1813) and *accD4*_*Msm*_ (MSMEG_6391) genes were PCR amplified on *M. smegmatis* chromosomal DNA template, using the primers described in Table S2. Each amplified fragment (1425 bp – *accD6*, 1629 bp – *accD5*, 1579 bp – *accD4*) was then cloned (*accD6, accD5* - XbaI/ClaI and *accD4* - XbaI/EcoRI) into pMVFAS2 Km vector under the control of P_*fasII*_ promoter. For the construction of pFD5Tb, the fragment (1680 bp) containing *accD5*_*Mtb*_ (Rv3280) gene was PCR amplified on a *M. tuberculosis* chromosomal DNA template and cloned under P_*fasII*_ promoter, using XbaI/ClaI restriction sites.

We used the Δ*accD6*_*Mtb*_-*attB::*P_*fasII*_-*D6*_*Mtb*_ *M. tuberculosis* mutant as the host for gene exchange experiments^14^. The native *accD6*_*Mtb*_ in this strain was deleted, and the only functional copy of this gene under P_*fasII*_ promoter was provided on pMV306 vector containing the hygromycin resistance gene. The pFD6Ms, pFD5Ms, pFD4Ms or pFD5Tb vectors were electroporated into Δ*accD6*_*Mtb*_-*attB::*P_*fasII*_-*D6*_*Mtb*_ competent cells. By plating the transformants onto 7H10/OADC agar containing kanamycin, the exchange mutants were selected, in which the *accD6*_*Mtb*_ was replaced by other gene with the same function because of the site-specific recombination in *attB*. The exchange mutant clones, grown in medium with kanamycin but not hygromycin, were subjected to genotype verification by Southern blot hybridization. For confirmation of *accD6*_*Mtb*_ – *accD6*_*Msm*_ exchange, the chromosomal DNA of the mutant as well as the plasmid DNA of the pFD6Ms vector (positive control) was digested with XbaI/BglII to release two *accD6*_*Msm*_ gene fragments (705 bp and 340 bp). The fragment of the same gene was PCR amplified using MsaccD6Xs and MsaccD6HXr primers and utilized as a hybridization probe. As an negative control (NC^1^) confirming the probe specificity, XbaI/HindIII-digested chromosomal DNA of the Δ*accD6*_*Mtb*_-*attB::*P_*fasII*_-*D6*_*Mtb*_ mutant was used (NC^1^ – confirms the inability of the probe to bind *accD6*_*Mtb*_ in gene exchange host strain). To exclude the possibility of non-specific probe binding to random sites in the *M. tuberculosis* genome, a NC^2^ negative control was prepared. For NC^2^, the XbaI/BglII-digested, wild-type *M. tuberculosis* chromosomal DNA was used. For Southern blot confirmation of *accD6*_*Mtb*_ – *accD5*_*Msm*_ exchange, the chromosomal DNA of the mutant as well as the plasmid DNA of the pFD5Ms vector (positive control) was digested with XbaI/BamHI to release the *accD5*_*Msm*_ gene fragment (823 bp). The fragment of the same gene was PCR amplified using MsaccD5flip-s and MsaccD5flip-r primers and utilized as a hybridization probe. The NC^1^ control was prepared as described above; for NC^2^, wild-type *M. tuberculosis* chromosomal DNA was digested with XbaI/BamHI. To confirm potential *accD6*_*Mtb*_ – *accD4*_*Msm*_ exchange, the chromosomal DNA of selected clones was digested with XbaI/EcoRI. The same enzymes were used to digest pFD4Ms plasmid DNA (positive control). As a probe, the *accD4*_*Msm*_ gene fragment was PCR amplified, using accD4Xbs and accD4Xbr primers. For NC^1^, the chromosome of the Δ*accD6*_*Mtb*_-*attB::*P_*fasII*_-*D6*_*Mtb*_ mutant digested with XbaI/EcoRI and XbaI/HindIII was used. For NC^2^, the wild-type *M. tuberculosis* chromosome digested with XbaI/EcoRI was used.

### Fatty acid extraction and analysis

Mycobacterial cultures were first mixed with [2-^14^C]acetate (specific activity, 45 to 60 mCi mmol^−1^; Perkin-Elmer) at 1 μCi ml^−1^ and then further incubated at 37 °C for 1 h (*M. smegmatis*) or 6 h (*M. tuberculosis*). The ^14^C-labeled cells were harvested by centrifugation at 2,000 × *g*, subjected to alkaline hydrolysis by incubation in 2 ml 15% tetrabutylammonium hydroxide (TBAH) (Sigma) at 100 °C overnight, and then mixed with 4 ml CH_2_Cl_2_, 300 μl CH_3_I (Sigma), and 2 ml H_2_O. After 1 h, the upper, aqueous phase was discarded, and the lower, organic phase was washed twice with water and dried. The lipids were extracted using diethyl ether, dried, and then resuspended in 200 μl CH_2_Cl_2_. Equal counts of the fatty acid methyl esters (FAMEs) and mycolic acid methyl esters (MAMEs) from the wild-type and mutant strains were applied to thin-layer chromatography (TLC) silica gel 60F_254_ plates (Merck) and developed in petroleum ether – acetone (19:1, vol/vol) or hexane – ethyl acetate (19:1, vol/vol).

## Additional Information

**How to cite this article**: Pawelczyk, J. *et al*. The influence of AccD5 on AccD6 carboxyltransferase essentiality in pathogenic and non-pathogenic *Mycobacterium. Sci. Rep.* **7**, 42692; doi: 10.1038/srep42692 (2017).

**Publisher's note:** Springer Nature remains neutral with regard to jurisdictional claims in published maps and institutional affiliations.

## Supplementary Material

Supplementary Information
