# Supplementary material for: The influence of AccD5 on AccD6 carboxyltransferase essentiality in pathogenic and non-pathogenic Mycobacterium
Source: Sci Rep. 2017 Feb 16;7:42692. doi: 10.1038/srep42692 (PMC5311964; doi:10.1038/srep42692)
Supplement: Supplementary Information [file srep42692-s1.pdf]

Supplementary Information

The influence of AccD5 on AccD6 carboxyltransferase essentiality in pathogenic and non-pathogenic *Mycobacterium*

Jakub Pawelczyk[1], Albertus Viljoen[2], Laurent Kremer[2,3] & Jaroslaw Dziadek[1]#

[1]Institute for Medical Biology, Polish Academy of Sciences, Lodz, Poland, and [2]Centre National de la Recherche Scientifique FRE 3689, Centre d'études d'agents Pathogènes et Biotechnologies pour la Santé, Université de Montpellier, Montpellier, France, and [3]INSERM, CPBS, 34293 Montpellier, France.

#Address correspondence to J. Dziadek, jdziadek@cbm.pan.pl, Tel.: 48 42 2723610

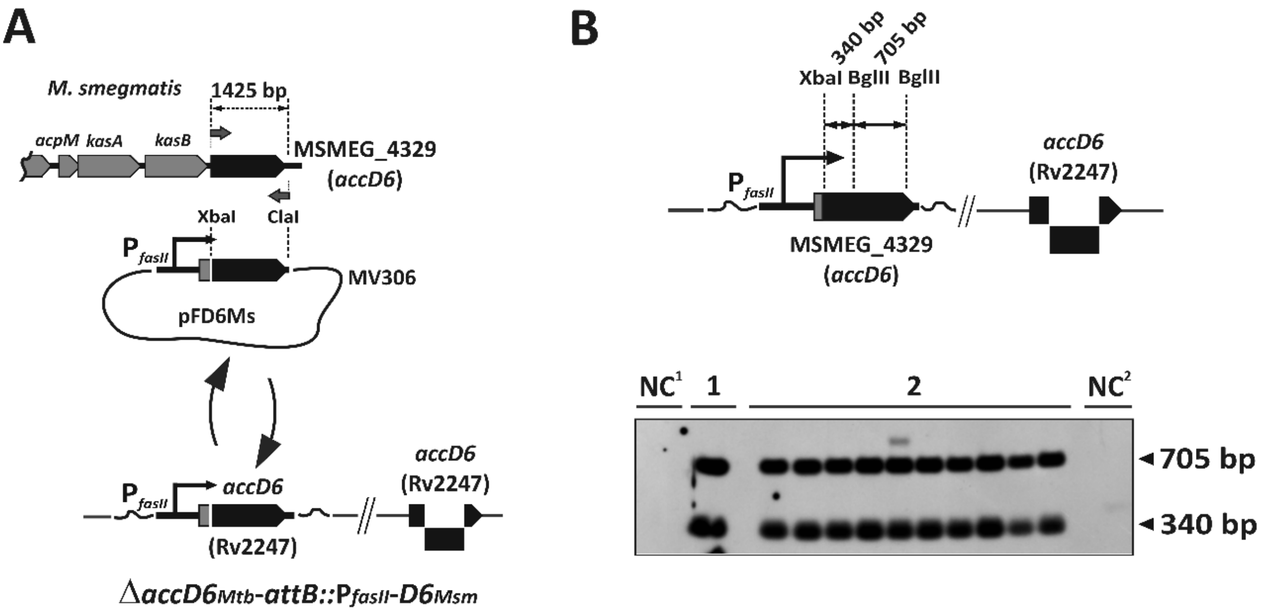

Fig. S1 A) The Δ*accD6*$_{Mtb}$*-attB::*P_{fasII}*-D6*$_{Msm}$ mutant construction scheme. The dashed line represents the size of the fragment that was amplified by PCR on chromosomal DNA of Msm using oligonucleotide primers MsaccD6flip-s and MsaccD6flip-r (gray arrows) and cloned under the control of Mtb P_{fasII} promoter into the pMV306 vector. MSMEG_4329 (accD6) effectively replaces the accD6$_{Mtb}$ gene in the Δ*accD6*$_{Mtb}$*-attB::*P_{fasII}*-D6*$_{Mtb}$ mutant, giving rise to the Δ*accD6*$_{Mtb}$*-attB::*P_{fasII}*-D6*$_{Msm}$ strain. **B) Southern blot confirmation of Δ*accD6*$_{Mtb}$*-attB::*P_{fasII}*-D6*$_{Msm}$ mutant genotype.** (top) Diagram showing the size of the restriction fragments (340 and 705 bp) after XbaI/BglII digestion. (bottom) Southern blot analysis confirming the integration of the *Msm accD6* gene into the chromosome of the Δ*accD6*$_{Mtb}$*-attB::*P_{fasII}*-D6*$_{Msm}$ mutant as the only functional copy of the *accD6*. Arrows indicate the size of the restriction fragments hybridized to a DNA probe, complementary to the *accD6*$_{Msm}$. **Lines: 1** – (positive control) XbaI/BglII-digested DNA of pFD6Ms vector, **2** – XbaI/BglII-digested chromosomal DNA of selected Δ*accD6*$_{Mtb}$*-attB::*P_{fasII}*-D6*$_{Msm}$ clones, **NC**^{1} – (negative control) XbaI/HindIII-digested chromosomal DNA of Δ*accD6*$_{Mtb}$*-attB::*P_{fasII}*-D6*$_{Mtb}$ strain (confirmation that the probe does not hybridize to *accD6*$_{Mtb}$), **NC**^{2} – (negative control) XbaI/BglII-digested chromosomal DNA of *Mtb* wild-type strain (exclusion of the probe non-specific binding to the random genomic regions of *Mtb* chromosome).

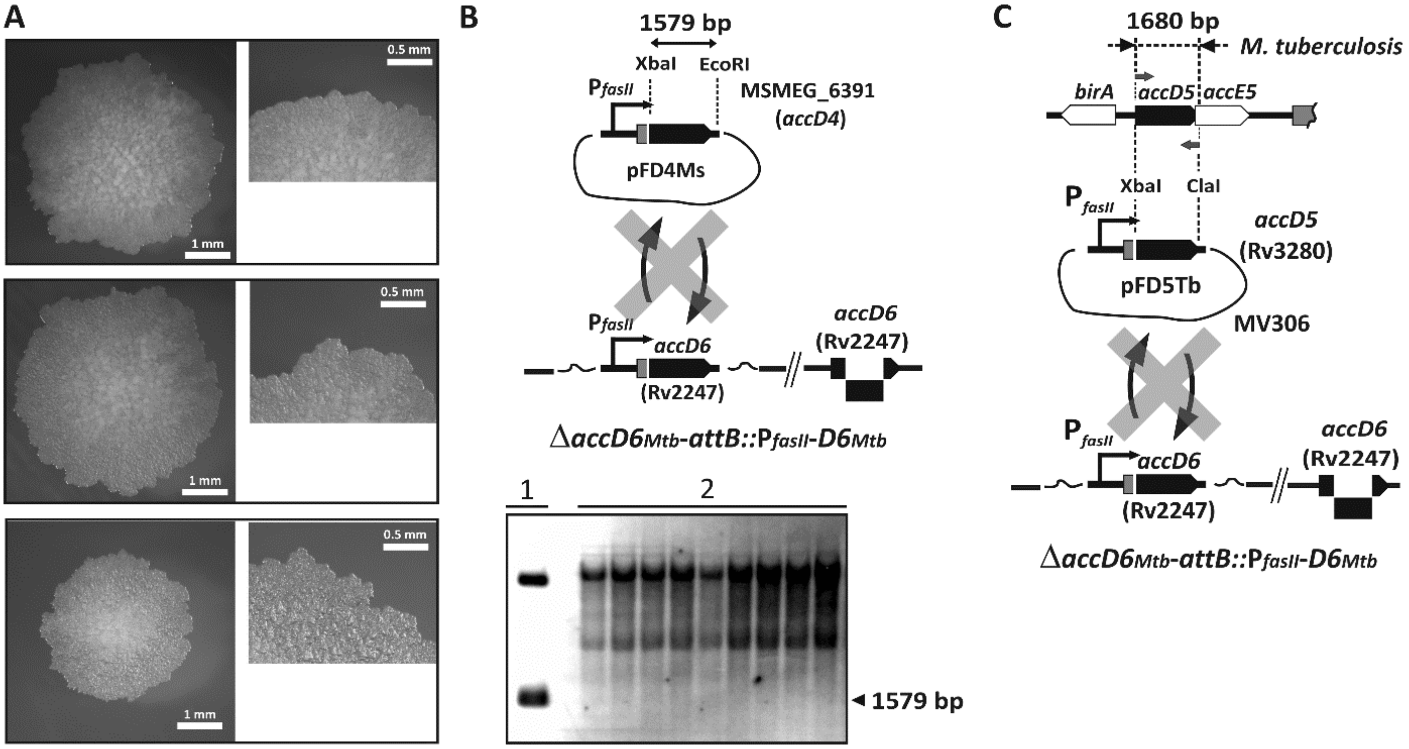

Fig. S2 A) Colony morphology of the *Mtb* wild-type strain (top), Δ*accD6*$_{Mtb}$*-attB::*P$_{fasII}$*-D6*$_{Msm}$, (middle) and Δ*accD6*$_{Mtb}$*-attB::*P$_{fasII}$*-D5*$_{Msm}$ mutant (bottom). Representative samples are shown in all panels. White bars represent 1 and 0.5 mm. **B) The study of the possibility of an exchange of functional acetyl-CoA carboxyltransferase gene - *accD6* (Rv2247) for MSMEG_6391 (*accD4*) in *Mtb* cell.** (top) Procedure used to exchange functional *accD6* in Δ*accD6*$_{Mtb}$*-attB::*P$_{fasII}$*-D6*$_{Mtb}$ for MSMEG_6391 (*accD4*$_{Msm}$), introduced into the mutant chromosome on a pFD4Ms vector. Long, thin arrow represent the XbaI/EcoRI restriction fragment of the *accD4*$_{Msm}$ gene, which should be the source of the Southern blot signal after hybridization with a probe that constitutes the PCR-amplified fragment of the same gene. (bottom) Southern blot analysis of mutant genotype. Lines represent **1** – plasmid DNA of pFD4Ms vector (positive control), **2** – chromosomal DNA of the nine clones that grew on selective medium after gene exchange procedure, with the black triangle indicating the position of the expected signal originating from the 1579 bp fragment of *accD4*$_{Msm}$. The hybridization signal was not detected in any of the analyzed DNA samples. **C) The study of the possibility of the exchange of *accD6* (Rv2247) for another copy *accD*5 (Rv3280) in *Mtb* Δ*accD6*$_{Mtb}$*-attB::*P$_{fasII}$*-D6*$_{Mtb}$ mutant.** The dashed line represents the size of the fragment that was PCR amplified on the chromosomal DNA of Mtb during pFD5Tb vector construction. After introducing pFD5Tb into the Δ*accD6*$_{Mtb}$*-attB::*P$_{fasII}$*-D6*$_{Mtb}$ mutant, all subsequently selected clones retained the functional *accD6* gene, which confirms that *Mtb* AccD5 (Rv3280) is unable to functionally replace the essential acetyl-CoA carboxyltransferase AccD6 (Rv2247), even after increasing the *accD5* gene copy number.

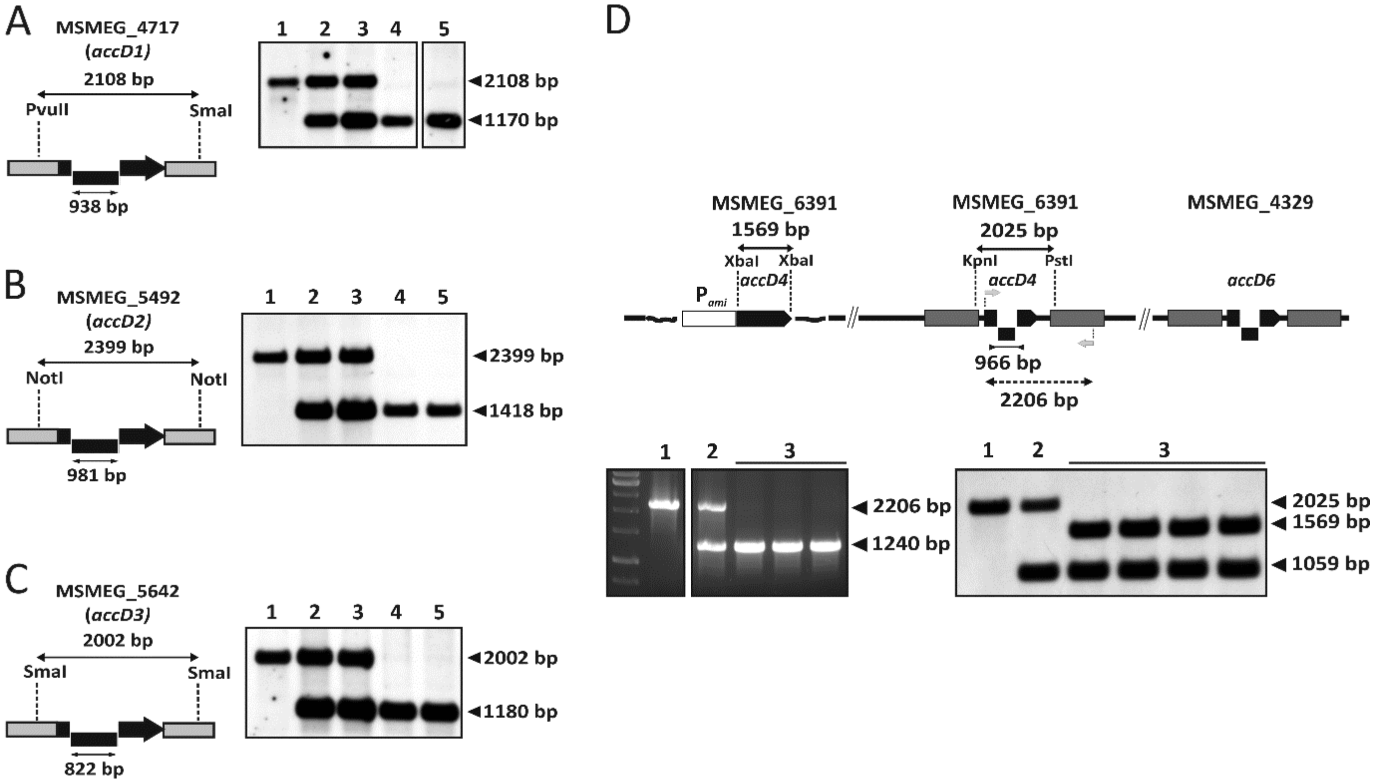

Fig. S3 A, B, C) Southern blot confirmation of the *Msm* knockout mutants genotype. (left) Diagrams showing the size of the restriction fragments (long thin arrows), deletion area (short thin arrows) and restriction endonucleases used. (right) Southern blot analysis confirming the deletion of **A)** – MSMEG_4717 (*accD1*$_{Msm}$), **B)** – MSMEG_5492 (*accD2*$_{Msm}$), **C)** – MSMEG_5642 (*accD3*$_{Msm}$). PCR-amplified fragments of three corresponding genes were used as the DNA hybridization probes. Lanes represent genomic DNA from the following *Msm* strains: **1** – *Msm* wild-type, **2** – **A)** *accD1* SCO, **B)** *accD2* SCO, **C)** *accD3* SCO, **3** – **A)** Δ*D6*;*D1* SCO, **B)** Δ*D6*;*D2* SCO, **C)** Δ*D6*;*D3* SCO, **4** - **A)** – Δ*accD1*, **B)** – Δ*accD2*, **C)** – Δ*accD3*, **5** - **A)** Δ*D6*;Δ*D1*, **B)** Δ*D6*;Δ*D2*, **C)** Δ*D6*;Δ*D3*. **D) Genotype analysis of Δ*D6*;Δ*D4-attB::*P$_{ami}$-*D4*$_{Msm}$ conditional mutant.** (top) Diagram showing the size of the restriction fragments (long, thin arrows), deletion area (short, thin arrow) and restriction endonucleases used for DNA sample digestion in Southern blot confirmation of Δ*D6*;Δ*D4-attB::*P$_{ami}$-*D4*$_{Msm}$ mutant genotype. The PCR-amplified fragment of the MSMEG_6391 (*accD4*$_{Msm}$) gene was used as the hybridization probe. Gray arrows represent binding sites for the two oligonucleotide primers accD4Xbs and MSaccD4GR4 used for PCR confirmation of the Δ*D6*;Δ*D4-attB::*P$_{ami}$-*D4*$_{Msm}$ mutant genotype. PCR product size is indicated by the dashed arrow. (bottom) PCR (left) and Southern blot (right) confirmation of the mutant genotype. Lanes represent the genomic DNA of **1** – *Msm* wild-type strain, **2** – Δ*D6*;*D4* SCO, **3** – three randomly selected Δ*D6*;Δ*D4-attB::*P$_{ami}$-*D4*$_{Msm}$ conditional mutants (among those selected as unable to grow in media without acetamide supplementation).

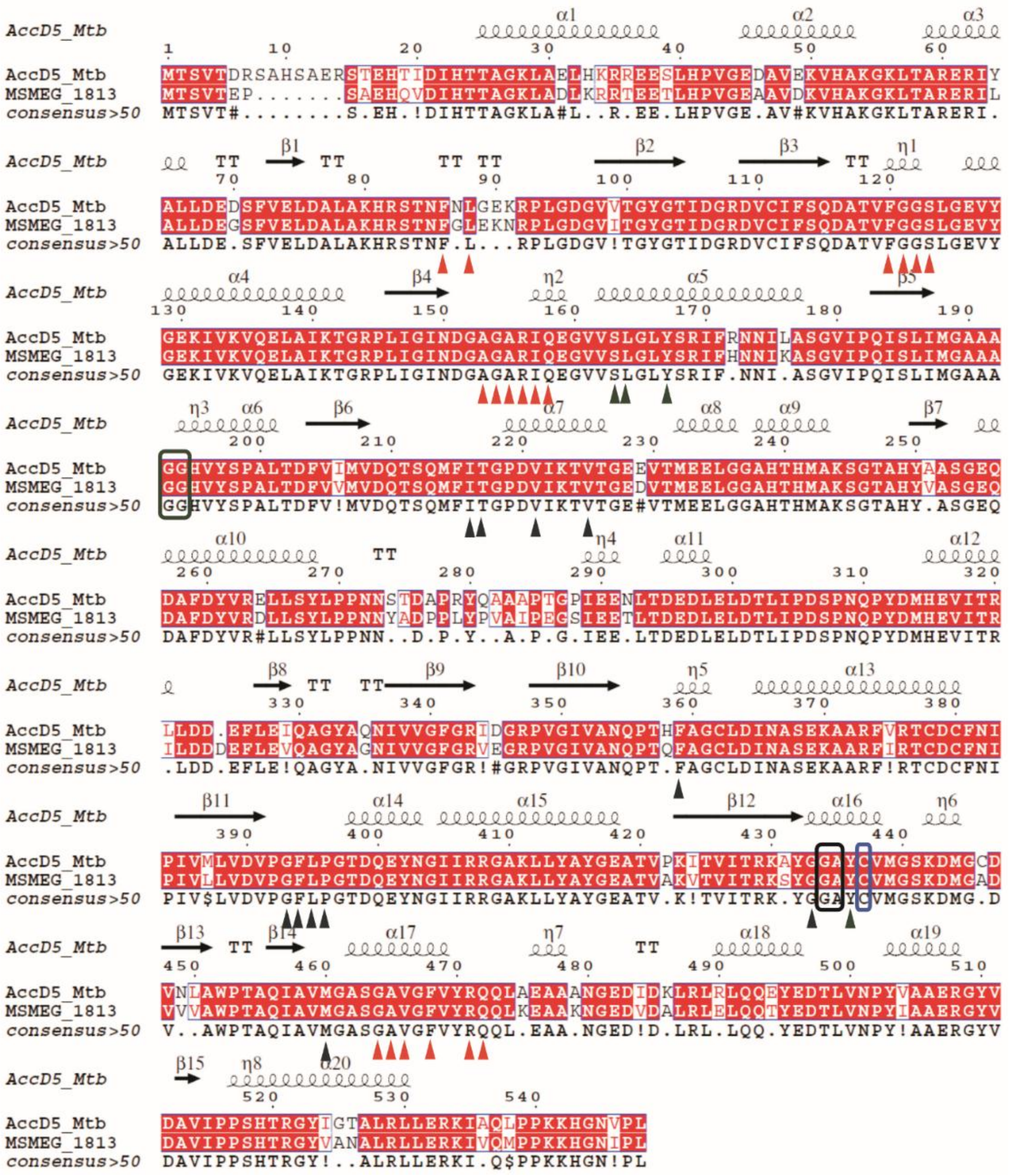

Fig. S4 Sequence alignment of AccD5 (Rv3280) and its homolog – MSMEG_1813 (AccD5$_{Msm}$). The secondary structure corresponding to AccD5 (Rv3280) (PDB entry 2a7s) is drawn at the top of the sequence alignment[1]. Helices, β-strands and turns are represented by coils, arrows and 'T's, respectively. Conserved residues are highlighted with a red background, and residues with similar chemical properties are colored red. Sequence alignment was performed using *ClustalW* [2]. The black frame indicates amino acids in active site that form hydrogen bonds with a biotin (Gly434; Ala435). Blue frame indicates amino acid that is directly responsible

for the active site substrate specificity (Cys437). Green frame indicates amino acids in active site that form hydrogen bonds with acyl carbonyl group (Gly193-194). Black triangles indicate amino acids that form a biotin binding pocket in active site. Orange triangles indicate amino acids that bind coenzyme A. Green triangles indicate amino acids that contribute to substrate binding.

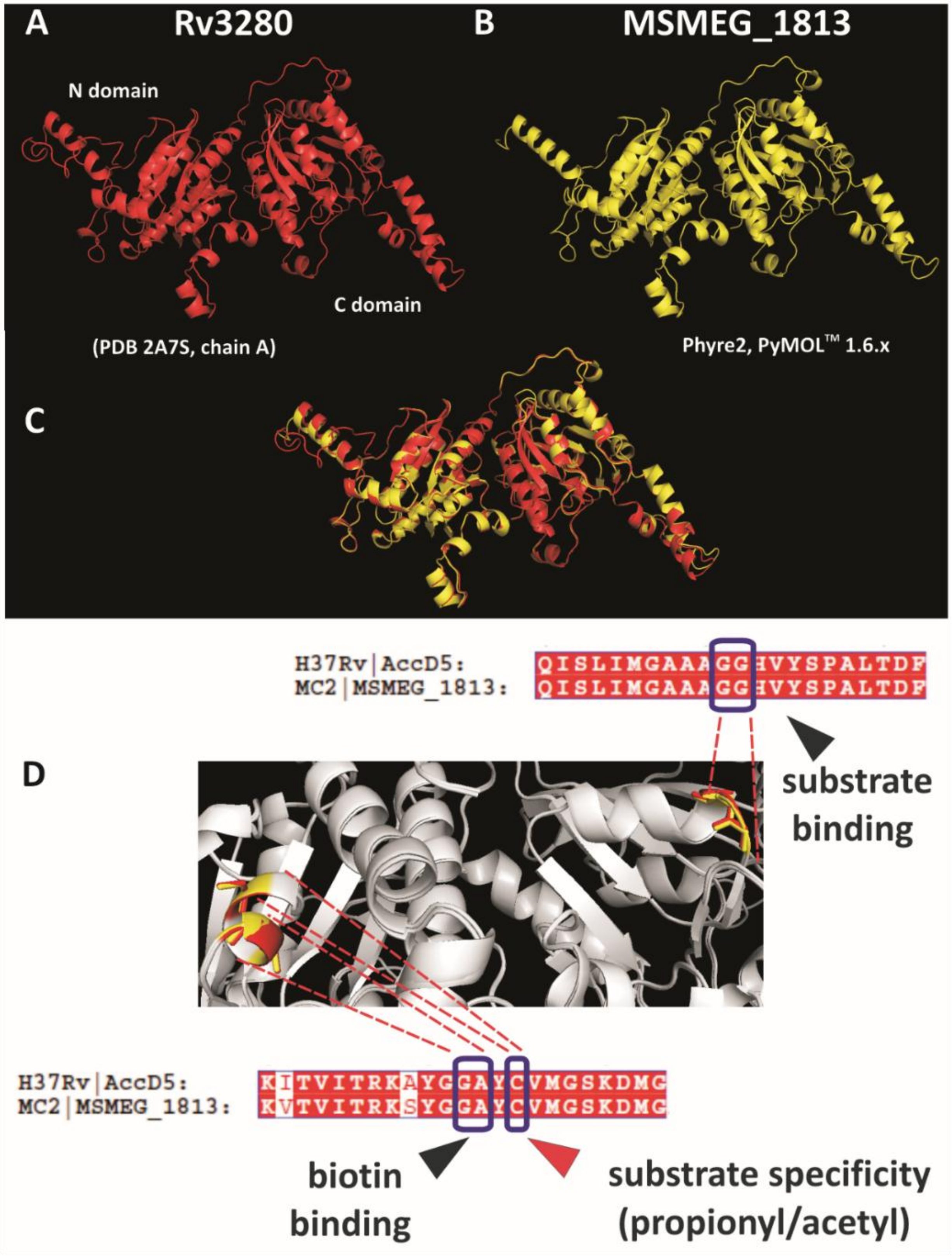

Fig. S5 Comparison of the *Mtb* and *Msm* AccD5 monomer structure. A) Ribbon diagram of the AccD5 (Rv3280) monomer structure, chain A (PDB entry 2a7s)[1]. **B)** Ribbon diagram of the MSMEG_1813 (AccD5$_{Msm}$) structure deduced using Phyre [3]. The positions of 98% of the amino acid residues were modeled with 90% confidence. The positions of 12 amino acid residues were modeled *ab initio*. **C)** Sequence-independent structural alignment of AccD5 (Rv3280) (red) and MSMEG_1813 (AccD5$_{Msm}$) (yellow). Alignment according

to the lowest RMSD value was performed using CEAlign of PyMOL™ 1.6.0.0 (The PyMOL™ Molecular Graphics System, Schrödinger, LLC.). **D)** Structural alignment of AccD5 (Rv3280) and MSMEG_1813 (AccD5$_{Msm}$) active site with particular emphasis on amino acids responsible for substrate specificity or biotin and substrate binding. The dashed lines indicate the amino acid position in the protein sequence.

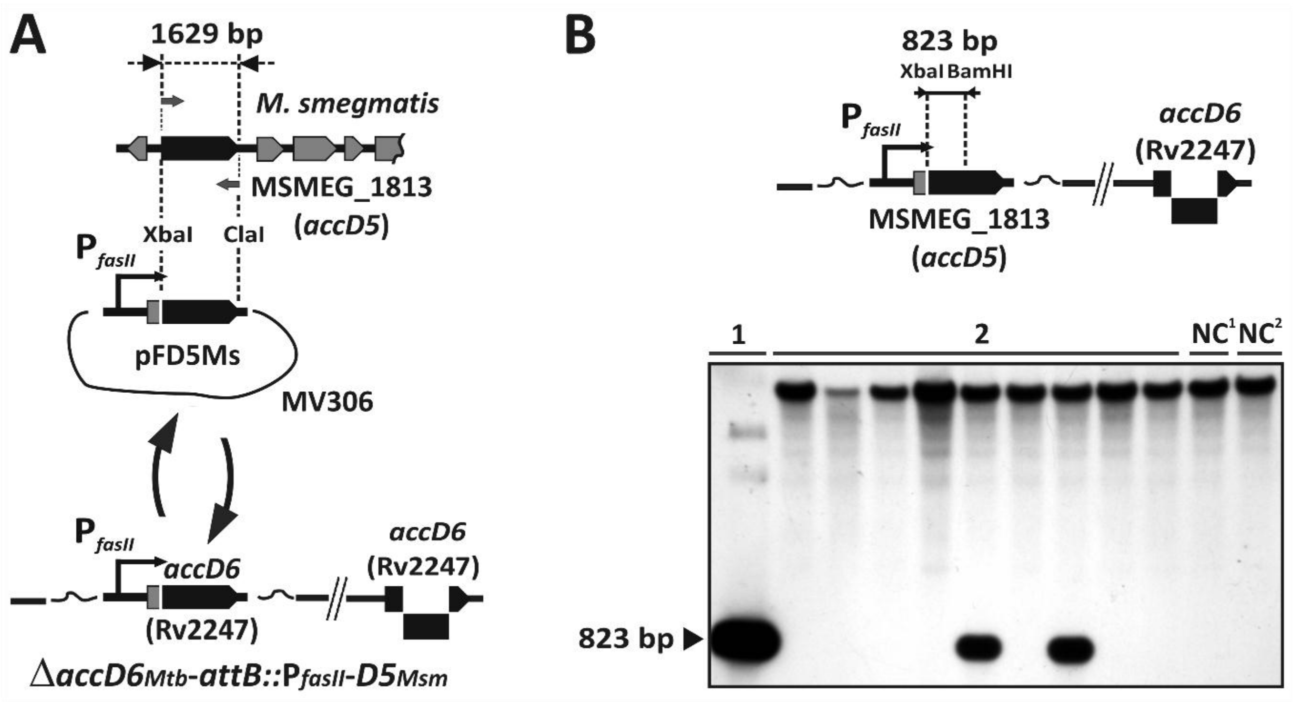

Fig. S6 A) The Δ*accD6*$_{Mtb}$-*attB::*P$_{fasII}$-*D5*$_{Msm}$ mutant construction scheme. The dashed line represents the size of the fragment that was amplified by PCR on the chromosomal DNA of Msm and cloned under the control of Mtb P$_{fasII}$ promoter into the pMV306 vector to construct pFD5Ms. MSMEG_1813 (accD5$_{Msm}$) effectively replaces the accD6$_{Mtb}$ gene in the Δ*accD6*$_{Mtb}$-*attB::*P$_{fasII}$-*D6*$_{Mtb}$ mutant, giving rise to the Δ*accD6*$_{Mtb}$-*attB::*P$_{fasII}$-*D5*$_{Msm}$ strain. **B) Southern blot confirmation of Δ*accD6*$_{Mtb}$-*attB::*P$_{fasII}$-*D5*$_{Msm}$ mutant genotype.** (top) Diagram showing the size of the restriction fragment (823 bp) after XbaI/BamHI digestion. (bottom) Southern blot analysis confirming the successful exchange of the accD6$_{Mtb}$ – functional acetyl-CoA carboxyltransferase gene for accD5$_{Msm}$ in two of the nine randomly selected Mtb mutant clones after selection. Black triangle indicate the position of the expected 823 bp Southern blot signal. Lines represent **1** – plasmid DNA of pFD5Ms vector (positive control), **2** – chromosomal DNA of the nine clones that grew on selective medium after the gene exchange procedure, **NC^1** – (negative control) XbaI/HindIII-digested chromosomal DNA of Δ*accD6*$_{Mtb}$-*attB::*P$_{fasII}$-*D6*$_{Mtb}$ strain (confirmation that the probe does not hybridize to *accD6* of *Mtb*), **NC^2** – (negative control) XbaI/BamHI-digested chromosomal DNA of *Mtb* wild-type strain (exclusion of the probe non-specific binding to the random genomic regions of *Mtb* chromosome). The expected hybridization signal was detected in DNA samples of two selected clones, confirming successful gene exchange. Two positive mutant clones were named Δ*accD6*$_{Mtb}$-*attB::*P$_{fasII}$-*D5*$_{Msm}$.

Plasmid	Description	Source or reference
**Cloning vectors**		
**pJET1.2/blunt**	PCR product cloning vector, Amp^R	Thermo Scientific
**p2NIL**	Recombination vector, nonreplicating in mycobacteria, Kan^R	(4)
**pGOAL17**	Source of PacI marker cassette (*sucB*, *lacZ*), Amp^R	(4)
**pJam2**	Shuttle vector carrying inducible P_{ami} promoter, Kan^R	(5)
**pMV306Km**	Mycobacterial integrating vector, Kan^R	Med.-Immune Inc.
**pMV306Hyg**	Mycobacterial integrating vector, Hyg^R	Med.-Immune Inc.
**Vectors used for gene replacement**		
**pJPD4Ms**	p2NIL-based recombination vector carrying the 5' end of *accD4*$_{Msm}$ and its upstream flanking sequence (GR1-GR2) (1158 bp) cloned next to the 3' end of *accD4*$_{Msm}$ and its downstream flanking sequence (GR3-GR4) (1202 bp), enriched with the PacI cassette from pGOAL17, Kan^R	This study
**pJPD3Ms**	p2NIL-based recombination vector carrying the 5' end of *accD3*$_{Msm}$ and its upstream flanking sequence (GR1-GR2) (1217 bp) cloned next to the 3' end of *accD3*$_{Msm}$ and its downstream flanking sequence (GR3-GR4) (1769 bp), enriched with the PacI cassette from pGOAL17, Kan^R	This study
**pJPD2Ms**	p2NIL-based recombination vector carrying the 5' end of *accD2*$_{Msm}$ and its upstream flanking sequence (GR1-GR2) (1497 bp) cloned next to the 3' end of *accD2*$_{Msm}$ and its downstream flanking sequence (GR3-GR4) 1414 bp), enriched with the PacI cassette from pGOAL17, Kan^R	This study
**pJPD1Ms**	p2NIL-based recombination vector carrying the 5' end of *accD1*$_{Msm}$ and its upstream flanking sequence (GR1-GR2) (1087 bp) cloned next to the 3' end of *accD1*$_{Msm}$ and its downstream flanking sequence (GR3-GR4) (1216 bp), enriched with the PacI cassette from pGOAL17, Kan^R	This study
**Vectors used for complementation**		
**pMVFAS2Km**	PCR fragment (1028 bp) carrying the promoter of the *M. tuberculosis* FAS II operon cloned into the NotI/XbaI site of the pMV306Km integrative vector	This study
**pFD6Ms**	pMVFAS2Km-based vector carrying the full-length sequence of *accD6*$_{Msm}$ (MSMEG_4329) (1425 bp) cloned into XbaI/ClaI site (P_{fasII}-*D6*$_{Msm}$)	This study
**pFD5Ms**	pMVFAS2Km-based vector carrying the full-length sequence of *accD5*$_{Msm}$ (MSMEG_1813) (1629 bp) cloned into XbaI/ClaI site (P_{fasII}-*D5*$_{Msm}$)	This study
**pFD5Tb**	pMVFAS2Km-based vector carrying the full-length sequence of *accD5*$_{Mtb}$ (Rv3280) (1680 bp) cloned into XbaI/ClaI site (P_{fasII}-*D5*$_{Mtb}$)	This study
**pFD4Ms**	pMVFAS2Km-based vector carrying the full-length sequence of *accD4*$_{Msm}$ (MSMEG_6391) (1579 bp) cloned into XbaI/EcoRI site (P_{fasII}-*D4*$_{Msm}$)	This study
**pAceD4Ms**	pMV306Hyg-based vector carrying the *accD4*$_{Msm}$ (MSMEG_6391) (1569 bp) under the control of the acetamidase (P_{ami}) promoter region (1500 bp) from pJam2 cloned into the HindIII/BamHI site	This study

Table S1 Plasmids used in this study.

Primer	Sequence 5' → 3'	Description
**Construction of gene replacement vectors**		
**MSaccD1GR1**<br>**MSaccD1GR2**	F: CGTCGCCGACCGGGACCTG<br>R: CAAGCTTAGCTTGCCCCGGCCGACG	GR1-GR2 PCR fragment of *accD1*$_{Msm}$ (1087 bp)
**MSaccD1GR3**<br>**MSaccD1GR4**	F: CAAGCTTGTCAAGGGAGCGCATTTCATCG<br>R: GGGGTACCTCGCCGAGATGGATGACGTTTC	GR3-GR4 PCR fragment of *accD1*$_{Msm}$ (1216 bp)
**MSaccD2GR1**<br>**MSaccD2GR2**	F: AACTGCAGGATCGGCCTACGTCGGGGTG<br>R: CGAAGCTTGAGCTTGGCCGTCATCACCG	GR1-GR2 PCR fragment of *accD2*$_{Msm}$ (1497 bp)
**MSaccD2GR3**<br>**MSaccD2GR4**	F: CGAAGCTTGCGGTGTGCTGTTCAGCGAGG<br>R: CGAAGCTTAACAGCTTGGCGCGCATGC	GR3-GR4 PCR fragment of *accD2*$_{Msm}$ (1414 bp)
**MSaccD3GR1**<br>**MSaccD3GR2**	F: GTCGTCGATCTGACCCGCGC<br>R: CGAAGCTTGACGGGGGAGTCCCATGAGG	GR1-GR2 PCR fragment of *accD3*$_{Msm}$ (1217 bp)
**MSaccD3GR3**<br>**MSaccD3GR4**	F: CGAAGCTTCGCGCTGGCCCGGTTCGGGC<br>R: GGGGTACCAACGGGGCGGGCGCGAGCTG	GR3-GR4 PCR fragment of *accD3*$_{Msm}$ (1769 bp)
**MSaccD4GR1**<br>**MSaccD4GR2**	F: AACTGCAGGACGTGCCGCCGCGTGAC<br>R: CAAGCTTCACCGCCGGGCTCCTTGG	GR1-GR2 PCR fragment of *accD4*$_{Msm}$ (1158 bp)
**MSaccD4GR3**<br>**MSaccD4GR4**	F: CAAGCTTCAACGAGGCGTCGGACAAGG<br>R: GGGGTACCGGAAACGTCTCCGCCAAGGG	GR3-GR4 PCR fragment of *accD4*$_{Msm}$ (1202 bp), *ΔD6;ΔD4-attB::*P$_{ami}$-*D4*$_{Msm}$ mutant genotype confirmation (MSaccD4GR4)
**Genotype confirmation**		
**MsD1co-s**<br>**MsD1co-r**	F: CGCCTTGTCATCGCATCGCG<br>R: CCGCGCGGTCGAGTAGTAGG	Confirmation of Δ*accD1*$_{Msm}$ genotype, Southern blot probe synthesis
**MsD2co-s**<br>**MsD2co-r**	F: ACTCCACGATCGATGCCACCTC<br>R: AGGCACATTCCGAGCACCGTG	Confirmation of Δ*accD2*$_{Msm}$ genotype, Southern blot probe synthesis
**MsD3co-s**<br>**MsD3co-r**	F: CACGGCTCGTGGACCTCATGGG<br>R: TTGTTGTTCGGCCATCTCGGCGGC	Confirmation of Δ*accD3*$_{Msm}$ genotype, Southern blot probe synthesis
**MsD4co-s**<br>**MsD4co-r**	F: GAAGCTGGAACTGGCCAAGGAGCC<br>R: AAGTTCAGGTCGGCCGAGAGCTGC	*accD4*$_{Msm}$ Southern blot probe synthesis
**MsaccD6Xs**<br>**MsaccD6HXr**	F: GCTCTAGAATGACGATCATGGCCCCCG<br>R: GCTCTAGAAAGCTTACAGCGGGATGTT CTTGTGG	*accD6*$_{Msm}$ Southern blot probe synthesis
**Construction of the complementation vectors**		
**accD4Xbs**<br>**accD4Xbr**	F: GCTCTAGAGTGACGAACAAGACCACCGCTG<br>R: GCTCTAGATCTTCGTGGGACGTCTACAGCG	pAceD4Ms vector construction, *accD4*$_{Msm}$ Southern blot probe synthesis, *ΔD6;ΔD4-attB::*P$_{ami}$-*D4*$_{Msm}$ mutant genotype confirmation (accD4Xbs)
**MsaccD6flip-s**<br>**MsaccD6flip-r**	F: GTCTAGAGTAAAGGAGGTTTGTATGACGATC ATGGCC<br>R: GATCGATCGCGCTGAGAAGTTACAGCGGG	pFD6Ms vector construction
**MsaccD5flip-s**<br>**MsaccD5flip-r**	F:GTCTAGAGTAAGAAAGGGAGTCCACATGACGAG CGTTACCGAGCCG<br>R: GATCGATCGTTCGCGCCGCTCACAG	pFD5Ms vector construction, *accD5*$_{Msm}$ Southern blot probe synthesis
**MsaccD4flip-s**<br>**MsaccD4flip-r**	F: GTCTAGACGTGACGAACAAGACCACCGCTGAAC<br>R: CGAATTCAGGATCGGGCCTCTTCGTGGG	pFD4Ms vector construction
**RvaccD5flip-s**<br>**RvaccD5flip-r**	F: GTCTAGACATGACAAGCGTTACCGACCGC<br>R: CATCGATCTCGACTCACAGGTTCGTTCCGC	pFD5Tb vector construction
**qRT-PCR primers**		
***accD4*$_{Msm}$**<br>**(MSMEG_6391)**	F: GCACTCGGAATGCCCTTCTTCTC<br>R: ACGAACAAGACCACCGCTGAACTC	*accD4*$_{Msm}$ expression analysis
sigA<br>**(MSMEG_2758)**	F: AGGGCTACAAGTTCTCGACCTACGCC<br>R: CCGAGCTTGTTGATCACCTCGACC	*sigA* (*mysA*) expression analysis

Table S2 Primers used for PCR amplification. Underlined regions indicate the restriction sites used during the cloning steps (ClaI ATCGAT, EcoRI GAATTC, HindIII AAGCTT, KpnI GGTACC, PstI CTGCAG, XbaI TCTAGA).

References

1. Lin, T.W., et al. Structure-based inhibitor design of AccD5, an essential acyl-CoA carboxylase carboxyltransferase domain of Mycobacterium tuberculosis. *Proc Natl Acad Sci USA* **103**, 3072-7 (2006).
2. Thompson, J.D., Higgins, D.G., & Gibson, T.J. CLUSTAL W: improving the sensitivity of progressive multiple sequence alignment through sequence weighting, position-specific gap penalties and weight matrix choice. *Nucleic Acids Res* **22**, 4673-4680 (1994).
3. Kelley, L.A., & Sternberg, M.J. Protein structure prediction on the Web: a case study using the Phyre server. *Nat Protoc* **4**, 363-71 (2009).
4. Parish, T., & Stoker, N.G. Use of a flexible cassette method to generate a double unmarked Mycobacterium tuberculosis tlyA plcABC mutant by gene replacement. *Microbiology* **146**, 1969-75 (2000).
5. Triccas, J.A., Parish, T., Britton, W.J., & Gicquel, B. An inducible expression system permitting the efficient purification of a recombinant antigen from *Mycobacterium smegmatis*. *FEMS Microbiol Lett* **167**, 151–156 (1998).
